# Association of bone-related biomarkers with femoral neck bone strength

**DOI:** 10.1186/s12891-022-05427-1

**Published:** 2022-05-21

**Authors:** Ning Xia, Yun Cai, Wei Wang, Chen Bao, Yunming Li, Qingyun Xie, Wei Xu, Da Liu

**Affiliations:** 1 Department of Orthopedics, The General Hospital of Western Theater Command, Rongdu Avenue No. 270, Chengdu, 610083 China; 2 Department of Critical Care Medicine, The Second Affiliated Hospital of Hainan Medical University, Haikou, 570311 China; 3 Key Laboratory of Applied Mechanics and Structural Safety of Sichuan Province, School of Mechanics and Aerospace Engineering, Southwest Jiaotong University, Chengdu, 610031 China; 4 Department of Information, Medical Support Center, The General Hospital of Western Theater Command, Rongdu Avenue No. 270, Chengdu, 610083 China

**Keywords:** Osteoporosis, Bone mineral density, Bone turnover markers, Bone strength, Biochemical markers

## Abstract

**Background:**

Femoral neck fractures are the worst consequence of osteoporosis (OP), and its early prevention and treatment have become a public health problem. This study aims to investigate the relationship of bone-related biomarkers, femoral neck bone mineral density (BMD) and maximum load (L_max_), selecting the indicator which can reflect femoral neck bone loss and reduced bone strength.

**Methods:**

A total of 108 patients were recruited from January 2017 to December 2019. Venous blood samples were collected from patients before total hip replacement, and femoral neck samples were collected during the surgery. Femoral neck BMD, femoral neck L_max_, bone-related markers (serum levels of bone turnover markers, protein expression of type I collagen (COL-I) and osteopontin (OPN) in femoral neck) were all measured and analyzed.

**Results:**

The expression of COL-I in femoral neck were significantly decreased, whereas other markers were all significantly increased with the decreasing of femoral neck BMD and L_max_ (*P* < 0.05). Among them, serum C-terminal telopeptide of type I collagen (CTX) levels and OPN expression of femoral neck were increased in osteopenia. In multiple linear regression analysis, CTX and OPN were both negatively correlated with femoral neck BMD and L_max_, and they were independent factors of femoral neck BMD and L_max_, whereas COL-I was independent factor affecting L_max_ (*P* < 0.05). Besides, CTX was negatively correlated with COL-I (*β* = -0.275, *P* = 0.012) and positively correlated with OPN (*β* = 0.295, *P* = 0.003).

**Conclusions:**

Compared with other indicators, serum CTX was more sensitive to differences in bone mass and bone strength of femoral neck, and could be considered as surrogate marker for OPN and COL-I.Early measurement of CTX could facilitate the diagnosis of osteopenia and provide a theoretical basis for delaying the occurrence of femoral neck OP and fragility fractures.

**Supplementary Information:**

The online version contains supplementary material available at 10.1186/s12891-022-05427-1.

## Introduction

Osteoporosis (OP) is a common systemic bone metabolic disease characterized by the reduction of bone mass and microarchitectural deterioration of bone tissue, which ultimately leads to increased bone fragility and fracture risk [1]. In recent years, with the increasing incidence of OP in the world, OP has become an important issue affecting human health [2]. Femoral neck osteoporotic fracture is a serious complication of OP, with the increasing of the aging population, the incidence of femoral neck osteoporotic fractures increases rapidly. Due to the long course, severe symptoms and poor prognosis, femoral neck osteoporotic fractures have attracted increasing attention [3]. Previous studies have shown that femoral neck fractures were in high risk of developing severe complications (e.g., deep vein thrombosis and bed sores) during bed rest, which not only deeply influence the life quality but also cause heavy economic burden on patients and society [4].

Currently, OP can be evaluated by bone mineral content (BMC) and bone strength. Bone strength can describe the maximum load carrying capacity of bone prior to failure directly. The estimation of whole bone strength via finite element analysis (FEA) has shown promise as an approach to assess fracture risk and treatment efficacy [5]. However, most finite element software of bone analyse is still in the trial stage, and FEA is very time consuming and complicated to operate. In 1994, the World Health Organization (WHO) recommended that bone mineral density (BMD) (BMD = BMC / projected bone area) can be the diagnostic criteria for OP. At present, dual-energy X-ray absorptiometry (DXA) is the reference standard and the most widely used method to assess BMD [6]. Due to the rapid detection, wide detection range, and non-invasiveness, DXA has been widely used in clinical practice [7]. However, DXA is susceptible to bone hyperplasia, fractures and extraosseous calcification at the measurement site. Furthermore, DXA cannot effectively distinguish between trabecular and cortical bone, the different metabolic rate between trabecular and cortical bone may affect BMD testing results [8]. The Fracture Risk Assessment Tool (FRAX) is the most widely used tool for fracture risk assessment, which computes the 10-year probability of major osteoporotic fracture and hip fracture [9]. FRAX improves fracture prediction over the BMD measurement alone, however, the FRAX performance of predicting fracture risk varies in different study populations. Hence, there is room for further improvement in fracture prediction [10].

Several studies have shown that many patients with fragility fractures have normal or slight low bone mass, and thus the diagnosis and evaluation of OP and fragility fractures through BMD alone are not comprehensive [11]. Previous studies have founded that bone-related biomarkers can well reflect bone turnover and the risk of OP [12].

Osteopenia is a critical transition stage from normal to osteoporosis, and thus the early detection of osteopenia patients could be of great importance for the timely prevention and treatment of osteoporosis [13]. However, many studies have only focused on the detection of OP, but few on the osteopenia. It is also unclear which indicator can better reflect the early bone loss and change of bone strength. Therefore, the purpose of this study was to find indicators, which can sensitively reflect the early change of bone mass and strength of femoral neck, through investigating the association of femoral neck BMD, femoral neck bone strength and bone-related biomarkers, further providing a theoretical basis for timely discovering the patients with femoral neck osteopenia and preventing the occurrence of femoral neck OP and femoral neck fragility fractures finally.

## Materials and methods

### Patients

Patients who underwent total hip replacement at the General Hospital of Western Theater Command due to femoral neck fracture, femoral head necrosis, or hip arthritis were recruited from January 2017 to December 2019. Age, height and body mass index (BMI) were all recorded. The inclusion criteria were as follows: a) total hip replacement surgery was performed; b) there was no obvious contraindications to surgery and no bone metabolic diseases (except OP); c) all patients had femoral neck BMD measured at the ipsilesional side; d) there was no history of mental illness; e) patients or their families signed an informed consent. Exclusion criteria were as follows: a) patients suffered from diseases affecting bone metabolism (e.g., diabetes, thyroid diseases, hyperparathyroidism and rheumatoid arthritis); b) patients received drugs that may affect bone metabolism within 3 months (e.g., glucocorticoids, thyroid drugs, vitamin D supplements and calcium supplements); c) patients who received previous antiosteoporotic treatment; d) severe organ dysfunction; e) severe deformity at the measurement site.

### Measurement of BMD

BMD (g/cm^2^) of the femoral neck were measured by DXA in one week before surgery (Lunar prodigy, GE Medical Systems, Madison, WI, USA). Based on the results of femoral neck BMD, all patients were divided into normal group (T-score ≤  -1.0), osteopenia group (T-score < -1.0 and > -2.5), OP group (at least one of the following criteria fulfilled: (a) T-score ≤  -2.5; (b) femoral neck fragile fracture) and severe OP group (fragility fracture with a T-score ≤  -2.5) according to the WHO diagnostic criteria and the Guidelines for the diagnosis and management of primary osteoporosis (2017).

### Analysis of bone-related biomarkers

#### Bone turnover markers (BTMs)

Blood samples were collected under fasting condition in the early morning in one week before surgery. All samples were immediately centrifuged, and serum was then separated and stored at -80 °C until analysis. Serum levels of osteocalcin (OC), type I procollagen N-terminal propeptide (PINP) and C-terminal telopeptide of type I collagen (CTX) were all measured by enzyme-linked immunosorbent assay (ELISA) (R&D Systems, Minneapolis, MN). The detection ranges of serum OC, PINP, and CTX were 2.00–64.00 ng/ml, 2.50–80.00 ng/ml, and 93.75–3000.00 pg/ml, respectively. All procedures were carried out strictly in accordance with the kit instructions.

#### Biochemical markers

Protein expression of osteopontin (OPN) and type I collagen (COL-I) in the femoral neck was analyzed by Western blot. During the total hip replacement, osteotomy was initially performed 0.5–1.0 cm above the minor trochanter, and then performed under the femoral head to obtain the femoral neck samples. After removing the surrounding soft tissues, the femoral neck was washed with phosphate-buffered saline (PBS) to remove blood and residues. Cortical bone was collected from the femoral neck region. The complete part of femoral neck was stored below -20 °C and used for compression test, the excess bone tissue of femoral neck was stored at –80 °C and then frozen in liquid nitrogen and ground into a powder for total protein extraction using the bone tissue protein extraction kit (Biomart, Beijing, China). The protein concentration was determined using the BCA protein assay kit (Beyotime, Shanghai, China). 30 μg of soluble protein were separated by 10% SDS‐polyacrylamide gel electrophoresis (SDS‐PAGE) and transferred to polyvinylidene fluoride (PVDF) membranes (Millipore Corp, Billerica, MA, USA). Then the PVDF membranes were blocked in 5% skim milk for 1 h at room temperature. Subsequently, the membranes were incubated with the following primary antibodies: anti-β-actin (1:10000, Immunoway, Plano, TX, USA), anti-Osteopontin (1:1000, Millipore Corp, Billerica, MA, USA), anti-Type I Collagen (1:500, Millipore Corp, Billerica, MA, USA) at 4 °C overnight. Prior to hybridisation with primary antibodies, membranes were cut at the each expected blots point. After washing with TBST, all membranes were incubated with secondary antibody (1:10000, Origene, Rockville, MD, USA) for 1 h at room temperature. After washing with TBST three times, PVDF membranes were detected by UVP Biochemi EC3 Imaging System (UVP, Upland, CA, USA) using chemiluminescence HRP substrate (Millipore Corp, Billerica, MA, USA). The ratio of the optical density of the target protein band to the reference protein band was calculated for statistical analysis.

#### Compression tests

**Fig. 1:**
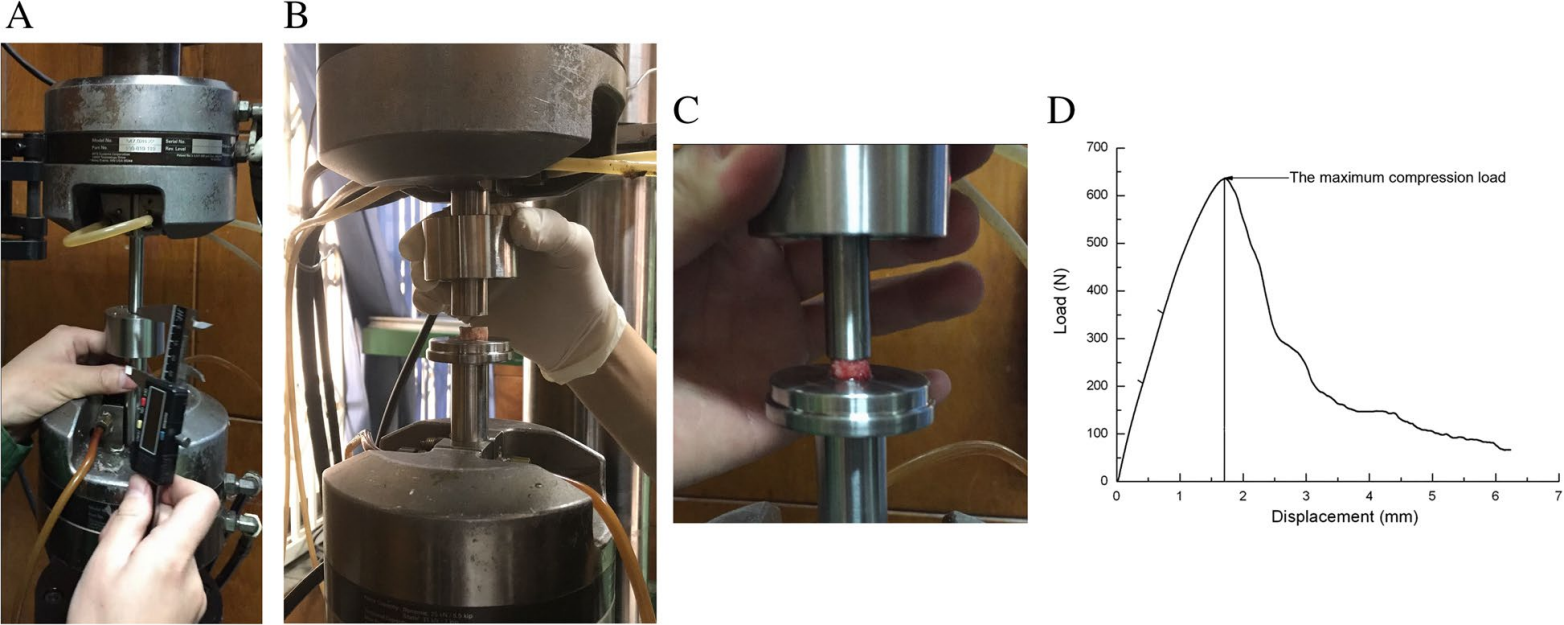
Axial compression experiment of the femoral neck. **A **The modulation of MTS model 809 axial/torsional testing system; **B **Placing specimens; **C **The femoral neck specimens after compressive test; **D **The load—displacement curve

Tensile experiments were conducted by using MTS model 809 axial/torsional testing system (MTS Systems Corp., USA). The MTS testing machine was equipped by an axial hydraulic actuator that had a 200 kN axial capacity*.* The cortical bone samples were dissected and cut in approximately 5 mm height and the superior and inferior planes were sanded to be parallel to each other. Before testing, samples were placed in the testing system and preloaded with a static preload of -10 N for 30 s. Subsequently, the compression test was performed with a 0.02 mm/s speed until the appearance of obvious peak, and then the maximum load (maximum load, L_max_) was automatically determined by the accompanying software (Fig. 1). All set of experiments were conducted at the room temperature of 23 ± 0.2 °C.

#### Statistical analysis

All data are consistent with a normal distribution and are presented as the mean ± SD. For quantitative data with normal distribution and homogeneity of variance (age, BMI, femoral neck BMD, protein expression of COL-I), one-way analysis of variance was performed, with differences among each group (normal group (T-score ≤  -1.0), osteopenia group (T-score < -1.0 and > -2.5), OP group (at least one of the following criteria fulfilled: (a) T-score ≤  -2.5; (b) femoral neck fragile fracture) and severe OP group (fragility fracture with a T-score ≤  -2.5)) assessed using a Bonferroni post hoc test. The data conform to a normal distribution, but they are not conformable in the homogeneity of the variance (femoral neck T-score and L_max_, serum levels of OC, PINP and CTX, protein expression of OPN) was analysed by the non-parametric Kruskal—Wallis test for statistically significant differences. A chi-squared test was used for categorical variables. Pearson correlation coefficients was used to analyze the relationship of the femoral neck BMD, femoral neck L_max_ and bone-related biomarkers. Multivariate linear regression analyses were performed to identify significant factors that affected the femoral neck BMD and L_max_. The receiver operating characteristic curve (ROC) analyse was used to evaluate the diagnostic value of indexes for femoral neck osteopenia and OP. *P* < 0.05 indicated that the difference was statistically significant. The statistical analysis was performed using IBM SPSS Statistics 25.0.

## Results

### General information of the patients

A total of 108 patients were selected in this study with an average age of 61.02 ± 10.84 years and an average BMI of 24.55 ± 3.41 kg/cm^2^. Subjects with severe osteoporosis were significantly older than those with either normal BMD or osteopenia (*P* < 0.0001). The average age of the subjects with osteopenia and osteoporosis were significantly higher compared with the normal BMD (*P* < 0.001). There were no significant differences in age among other groups (*P* > 0.05). The femoral neck T-score, BMD and L_max_ in the severe OP group were all significantly lower than those in the normal, osteopenia and OP groups (*P* < 0.0001). The femoral neck T-score, BMD and L_max_ in the OP and osteopenia groups were significantly lower than those in the normal group (*P* < 0.05). The differences among the other groups were not statistically significant (*P* > 0.05). There were no significant differences in the ratio of men to women and BMI among the groups (*P* > 0.05) (Table 1).

**Table 1:** Comparison of general clinical information of the patients in each group

Parameters	Normal ^a^ (*n* = 27, fragility fracture *n* = 0)	Osteopenia ^b^ (*n* = 27, fragility fracture *n* = 0)	Osteoporosis ^c^ (*n* = 27, fragility fracture *n* = 15)	Severe osteoporosis ^d^ (*n* = 27, fragility fracture *n* = 27)	*P*-value
Gender (male/female)	6/21	7/20	6/21	5/22	0.934
Age (years)	51.48 ± 10.71	60.30 ± 6.03^*^	63.00 ± 9.05^*^	69.30 ± 8.97^*#^	< 0.0001
BMI (kg/cm^2^)	24.85 ± 3.57	24.76 ± 3.89	24.88 ± 2.78	23.72 ± 3.37	0.545
Femoral neck BMD (g/cm^2^)	0.94 ± 0.12	0.80 ± 0.10^*^	0.77 ± 0.09^*^	0.61 ± 0.10^*#▲^	< 0.0001
Femoral neck T-score	0.00 ± 0.90	-1.50 ± 0.48^*^	-1.75 ± 0.75^*^	-2.91 ± 0.40^*#▲^	< 0.0001
Femoral neck L_max_ (KN)	3.00 ± 0.52	1.67 ± 0.46^*^	1.12 ± 0.26^*^	0.59 ± 0.19^*#▲^	< 0.0001

Data are presented as the mean ± SD for age, BMI, femoral neck BMD, femoral neck T-score and femoral neck L_max_

^a^ T-score ≥ -1.0

^b^ T-score < -1.0 and > -2.5

^c^ At least one of the following criteria fulfilled: (a) T-score ≤ -2.5; (b) femoral neck fragile fracture)

^d^ Fragility fracture with a T-score ≤ -2.5

^*^P < 0.05, compared with normal group

^#^*P* < 0.05, compared with osteopenia group

^▲^*P* < 0.05, compared with osteoporosis group

*BMI* body mass index, *BMD* bone mineral density

### Comparison of serum BTMs

Serum CTX and OC levels in the severe OP group were significantly higher than that in the normal and osteopenia groups, the levels of serum PINP were significantly increased compared with the normal group (*P* < 0.0001). Serum OC levels in the OP group were significantly increased than those in the normal and osteopenia groups, the levels of serum CTX and PINP were significantly increased compared with the normal group (*P* < 0.0001). Serum CTX levels in the osteopenia group were significantly higher than that in the normal group (*P* < 0.0001). The differences of the other groups were not statistically significant (*P* > 0.05) (Table 2).

**Table 2:** Comparison of serum BTMs levels in each group

Parameters	Normal ^a^ (*n* = 27)	Osteopenia ^b^ (*n* = 27)	Osteoporosis ^c^ (*n* = 27)	Severe osteoporosis ^d^ (*n* = 27)	*P*-value
CTX (ng/ml)	0.29 ± 0.14	0.52 ± 0.16^*^	0.65 ± 0.20^*^	0.78 ± 0.23^*#^	< 0.0001
PINP (ng/ml)	44.00 ± 12.55	48.30 ± 15.95	62.11 ± 21.04^*^	65.04 ± 23.73^*^	< 0.0001
OC (ng/ml)	8.33 ± 4.18	10.41 ± 4.67	17.70 ± 8.26^*#^	18.75 ± 10.45^*#^	< 0.0001

Data are presented as the mean ± SD for CTX, PINP and OC

^a^ T-score ≥ -1.0

^b^ T-score < -1.0 and > -2.5

^c^ At least one of the following criteria fulfilled: (a) T-score ≤ -2.5; (b) femoral neck fragile fracture)

^d^ Fragility fracture with a T-score ≤ -2.5

^*^P < 0.05, compared with normal group

^#^*P* < 0.05, compared with osteopenia group

^▲^ P < 0.05, compared with OP group

*CTX C*-terminal telopeptide of type I collagen, *PINP* type I procollagen N-terminal propeptide, *OC* osteocalcin

### Comparison of OPN and COL-I protein expression

**Fig. 2:**
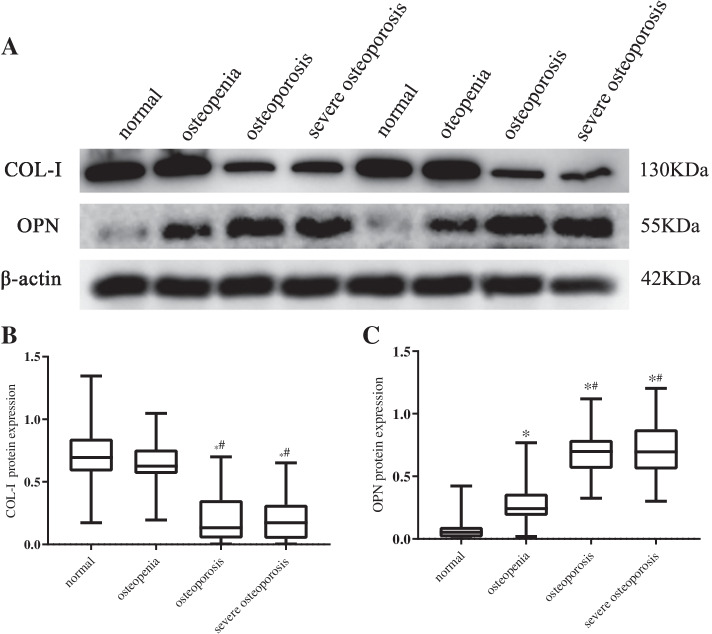
Expression of COL-I and OPN in femoral neck with different femoral neck BMD. **A** Western blot analysis of COL-I and OPN expression in different groups; **B** Comparison of COL-I expression among different groups; **C **Comparison of OPN expression among different groups. ^a^ T-score ≥ -1.0, ^b^ T-score < -1.0 and > -2.5, ^c^ At least one of the following criteria fulfilled: (a) T-score ≤ -2.5; (b) femoral neck fragile fracture), ^d^ Fragility fracture with a T-score ≤ -2.5. ^*^P < 0.05, compared with normal group; ^#^*P* < 0.05, compared with osteopenia group. COL-I type I collagen, OPN osteopontin

Expression of OPN in the severe OP and OP groups was significantly increased compared with the normal and osteopenia groups (*P* < 0.05). Expression of COL-I in the severe OP and OP groups was significantly lower than that in the normal and osteopenia groups (*P* < 0.05). Expression of OPN in the osteopenia group was significantly increased compared with the normal group (*P* < 0.05). The differences of the other groups were not statistically significant (*P* > 0.05) (Fig. 2).

### Correlation of the femoral neck BMD, femoral neck L_max_ and other parameters

Pearson correlation analysis results showed that femoral neck BMD and femoral neck L_max_ were negatively correlated with serum BTMs and femoral neck OPN (femoral neck BMD: *r* = -0.531 ~ -0.301, *P* < 0.01; femoral neck L_max_: *r* = -0.644 ~ -0.353, *P* < 0.0001), whereas them were both positively correlated with femoral neck COL-I (femoral neck BMD: *r* = 0.402, *P* < 0.0001; femoral neck L_max_: *r* = 0.527, *P* < 0.0001). Among these markers, serum CTX had the strongest correlation with femoral neck BMD and femoral neck L_max_ (femoral neck BMD: *r* = -0.531, *P* < 0.0001, femoral neck L_max_: *r* = -0.660, *P* < 0.0001) (Table 3).

**Table 3:** Pearson correlation coefficients of femoral neck BMD, femoral neck L_max_ and other parameters

Parameters		Femoral neck BMD	Femoral neck L_max_	OC	PINP	CTX	COL-I
Femoral neck L_max_	*r*	0.655					
	*P*	< 0.0001					
OC	*r*	-0.352	-0.435				
	*P*	< 0.0001	< 0.0001				
PINP	*r*	-0.301	-0.353	0.349			
	*P*	0.002	< 0.0001	< 0.0001			
CTX	*r*	-0.531	-0.660	0.394	0.497		
	*P*	< 0.0001	< 0.0001	< 0.0001	< 0.0001		
COL-I	*r*	0.402	0.527	-0.246	-0.259	-0.401	
	*P*	< 0.0001	< 0.0001	0.010	0.007	< 0.0001	
OPN	*r*	-0.481	-0.644	0.419	0.355	0.487	-0.415
	*P*	< 0.0001	< 0.0001	< 0.0001	< 0.0001	< 0.0001	< 0.0001

*CTX C*-terminal telopeptide of type I collagen, *PINP* type I procollagen N-terminal propeptide, *OC* osteocalcin, *OPN* osteopontin, *COL*- *I* type I collagen

### Multiple linear regression analysis

The femoral neck BMD and L_max_ were used as dependent variable, respectively, and serum BTMs, femoral neck biochemical markers, age, BMI and gender were all included as independent variables in multivariate linear regression models. There were no interaction among variables presented in Table 4 was found in multiple linear regression analyses. Multivariate linear regression analysis revealed that the femoral neck BMD was negatively correlated with femoral neck OPN protein expression (*β* = -0.220, *P* = 0.025) and serum CTX levels (*β* = -0.331, *P* = 0.001). After adjusting age, gender and BMI, we found that femoral neck BMD was negatively correlated with CTX (*β* = -0.284, *P* = 0.004) and OPN (*β* = -0.203, *P* = 0.037) (Table 4).

**Table 4:** Multiple linear regression analysis of femoral neck BMD, femoral neck L_max_ and other parameters

Parameters	Femoral neck L_max_			Femoral neck BMD		
	*S.E*	Standardized Coefficients Beta	*P*-value	*S.E*	Standardized Coefficients Beta	*P*-value
OC	0.008	-0.068	0.301	0.002	-0.036	0.684
PINP	0.003	0.101	0.138	0.001	0.083	0.361
CTX	0.283	-0.357	< 0.0001	0.057	-0.284	0.004
OPN	0.221	-0.355	< 0.0001	0.045	-0.203	0.037
COL-I	0.208	0.149	0.024	0.042	0.100	0.255
adjusted-R^2^		0.668			0.404	

Adjusted for age, BMI and sex

*CTX C*-terminal telopeptide of type I collagen, *PINP* type I procollagen N-terminal propeptide, *OC* osteocalcin, *OPN* osteopontin, *COL*- *I* type I collagen

As shown in Table 4, femoral neck L_max_ was negatively correlated with OPN (*β* = -0.355, *P* < 0.0001) and CTX (*β* = -0.357, *P* < 0.0001), whereas it was positively correlated with COL-I (*β* = 0.149, *P* = 0.024) after adjusting age, gender and BMI. Among above indexes, CTX had the strongest correlation with femoral neck BMD and femoral neck L_max_ (*P* < 0.01).

The association of BTMs and biochemical markers was further performed (Table 5). After adjusting age, gender and BMI, we found that CTX was negatively correlated with COL-I (*β* = -0.275, *P* = 0.012) and positively correlated with OPN (*β* = 0.295, *P* = 0.003).

**Table 5:** Multiple linear regression analysis between serum BTMs and femoral neck biochemical markers

Parameters	OPN			COL-I		
	*S.E*	Standardized Coefficients Beta	*P*-value	*S.E*	Standardized Coefficients Beta	*P*-value
OC	0.004	0.192	0.039	0.004	-0.041	0.686
PINP	0.002	0.042	0.664	0.002	< 0.0001	0.998
CTX	0.123	0.295	0.003	0.131	-0.275	0.012
adjusted-R^2^		0.319			0.180	

Adjusted for age, BMI and sex

*CTX C*-terminal telopeptide of type I collagen, *PINP* type I procollagen N-terminal propeptide, *OC* osteocalcin, *OPN* osteopontin, *COL*- *I* type I collagen

### ROC curve analysis

**Fig. 3:**
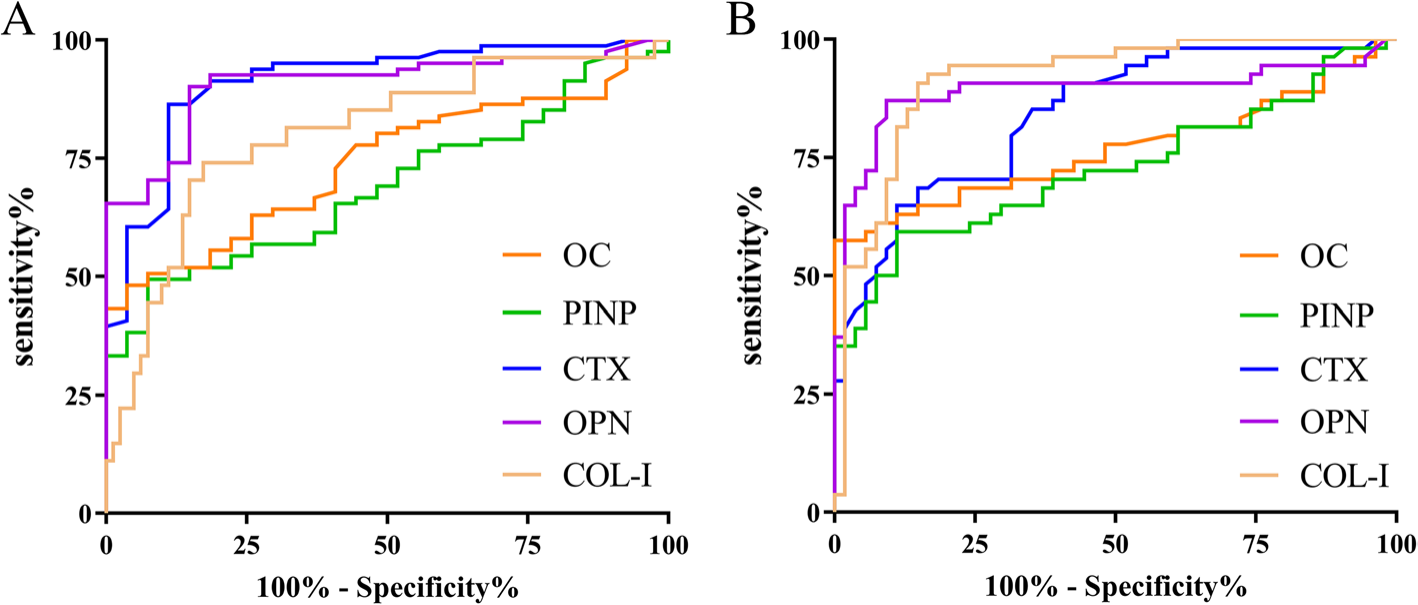
ROC analysis for each marker. **A **ROC curve of each marker for diagnosis of femoral neck osteopenia; **B **ROC curve of each marker for diagnosis of femoral neck osteoporosis. CTX C-terminal telopeptide of type I collagen, PINP type I procollagen N-terminal propeptide, OC osteocalcin, OPN osteopontin, COL- I type I collagen

To assess the potential diagnostic value of bone-related biomarkers in osteopenia from normal patients, the ROC curve analysis was performed. As shown in Table 6 and Fig. 2, the AUC of CTX and OPN were significantly higher than PINP and OC (*P* < 0.05). The differences of other markers were not statistically significant (*P* > 0.05). Furthermore, ROC analysis of each marker in estimating OP patients from normal and osteopenia patients was further performed. We found that the AUC of OPN and COL-I were greater than PINP and OC (*P* < 0.05). The differences of other markers were not statistically significant (*P* > 0.05) (Table 7, Fig. 3).

**Table 6:** The value of each marker in diagnosing femoral neck osteopenia

Parameters	AUC	*95% CI*	*S.E*	Cut-off	Sensitivity (%)	Specificity (%)	*P*-value
OC	0.741	0.648 ~ 0.834	0.047	15.74	0.481	0.963	< 0.0001
PINP	0.695	0.595 ~ 0.794	0.051	58.99	0.494	0.926	0.003
CTX	0.914	0.852 ~ 0.976	0.032	0.41	0.864	0.889	< 0.0001
COL-I	0.802	0.701 ~ 0.904	0.052	0.63	0.975	0.963	< 0.0001
OPN	0.908	0.851 ~ 0.965	0.029	0.14	0.901	0.852	< 0.0001

*AUC* the area under the curve, *CTX C*-terminal telopeptide of type I collagen, *PINP* type I procollagen N-terminal propeptide, *OC* osteocalcin, *OPN* osteopontin, *COL- I* type I collagen

**Table 7:** The value of each marker in diagnosing osteoporosis

Parameters	AUC	*95% CI*	*S.E*	Cut-off	Sensitivity (%)	Specificity (%)	*P*-value
OC	0.767	0.672 ~ 0.862	0.048	16.98	0.611	0.926	< 0.0001
PINP	0.726	0.628 ~ 0.824	0.050	61.26	0.593	0.889	< 0.0001
CTX	0.842	0.769 ~ 0.914	0.037	0.59	0.685	0.852	< 0.0001
COL-I	0.916	0.861 ~ 0.972	0.028	0.46	0.852	0.907	< 0.0001
OPN	0.891	0.819 ~ 0.962	0.036	0.44	0.870	0.907	< 0.0001

*AUC* the area under the curve, *CTX C*-terminal telopeptide of type I collagen, *PINP* type I procollagen N-terminal propeptide, *OC* osteocalcin, *OPN* osteopontin, *COL*- *I* type I collagen

## Discussion

Along with an increasing aging population, the number of patients with OP and fragility fractures gradually increase. It is estimated that there will be 5.99 million patients with osteoporotic fractures by 2050 in China [14]. Recently, femoral neck fragility fractures have received more and more attention due to the high rate of morbidity and mortality [4].

DXA is currently the golden standard for the diagnosis of OP, but it is not sensitive to monitor early changes of BMD, the change of bone loss can be identified by DXA only when bone loss reaches a certain level [15]. Moreover, OP is characterized by insidious onset and slow progression and is easily to be ignored, several patients already missed the best opportunity for treatment before diagnosed, leading to a poor prognosis.

Femoral neck L_max_ is defined as the maximum force that femoral neck can tolerate before fracture, and it is an important quantity for the mechanical property [16]. The early change of bone strength not only may give insight into the bone quality directly, but also contribute to the development of rational programs of prevention and treatment. However, the measurement of bone strength need be performed by vitro, it would enhance the difficulty of work. Due to the convenience, non-invasion and rapidity of serological examination, it is of significance to select an indicator which has stronger relationship with bone strength among the known biomarkers.

Bone-related biomarkers are used wildly in clinical practice. However, they are more used to evaluate the effect of drugs against osteoporosis treatment than reflect early changes in bone mass and bone strength and do not better inform medical staff about the bone quality or guide patients to change their lifestyle early in preventing bone loss. Furthermore, there are many types of bone-related biomarkers, and some of these indexes come at higher test prices [17]. If the indicator with greater sensitivity can be screened, the economic burden of patients can be reduced to a certain extent. Finally, the current research has mostly focused on the correlation between bone-related biomarkers and bone mineral density, and has not been extensively studied on bone strength. BMD only explain about 60—70% of the variation in bone strength [18], and mere correlation analysis of biomarkers with BMD does not well reflect changes in bone strength, which may be detrimental to our clinical guidance more comprehensively.

PN is one of the main non-collagen proteins in bone tissue, it is mainly involved in inhibiting bone mineral deposition and accelerating bone loss [19]. Multiple studies have shown that OPN is significantly increased in patients with OP compared with patients with normal bone mass [20]. COL-I is the major components of organic bone matrix and has an important effect on bone mechanical strength. Haynl et al. [21] showed that the decline of COL-I protein expression can lead to an increase in the risk of OP. Although several studies have shown that OPN and COL-I both play an important role in the incidence and development of OP [22–24], there are few studies on the changes of OPN and COL-I protein expression in the femoral neck under different femoral neck BMD. The effects of OPN and COL-I on the bone loss and reduced bone strength of femoral neck are still unclear.

In our study, we showed that COL-I protein expression of femoral neck in the severe OP and OP groups was significantly lower than that in both normal group and osteopenia group (*P* < 0.05). Furthermore, we found that protein expression of COL-I in femoral neck was positively correlated with the femoral neck L_max_ (*β* = 0.149, *P* = 0.024), indicating that the reduced bone strength of femoral neck is related to COL-I protein expression, and the decreased expression of COL-I may be the cause of femoral neck OP and femoral neck fragility fractures. At the same time, we found that with the gradual decreases of femoral neck BMD and L_max_, expression of OPN gradually increased (*P* < 0.05). Furthermore, OPN was negatively correlated with the femoral neck BMD and L_max_ (*P* < 0.05), indicating that the increased OPN might be the independent risk factor for the decline of femoral neck bone mass and bone strength. In addition, we are currently analyzing the interactions among the biochemical markers, spatial structure and biomechanical properties of femoral neck in order to fully elucidate the impact of the biochemical markers on the overall biomechanics of the femoral neck and their relationship with femoral neck fractures.

Serum BTMs can reflect the overall status of bone metabolism and detect the early changes of bone mass, they play an important role in guiding OP clinical diagnosis and treatment [25, 26]. The results of our study showed that serum CTX, PINP, and OC levels in the severe OP and OP groups were significantly higher than those in the normal group (*P* < 0.0001). These results are consistent with previous studies [27] showing that serum CTX, PINP and OC levels can reflect the changes in bone metabolism and bone mass, facilitating the early diagnosis and treatment of OP. We also found that serum CTX levels has changed significantly at the stage of femoral neck osteopenia, which indicates that serum CTX are more sensitive to the decline of femoral neck BMD, the measurement of serum CTX is conducive to early prevention and treatment of femoral neck OP. There are also some studies showing that with the decreases of BMD, serum OC levels gradually decrease [28]. Julien et al. [29] found that there was no significant difference in serum OC levels between OP patients and non-OP patients (*P* > 0.05). The discrepancy could be considered that all patients in previous studies were male who had relatively high peak bone mass, slow bone loss, and low conversion state of bone metabolism. Thus, the upward trend of serum OC levels in male patients is not significant.

We find that serum BTMs can reflect the change of femoral neck bone mass and bone strength, but there are multiple BTMs and which indicator can better reflect the early reduction of femoral neck bone mass and bone strength is still unknown. So, further analyses were carried out. After adjusting age, BMI and gender, multivariate linear regression analysis revealed that serum CTX was negatively correlated with femoral neck BMD and L_max_ (*P* < 0.01), indicating that the increased serum CTX was the independent risk factor for the reduced, early measurement of CTX can better reflect the loss of femoral neck bone mass and bone strength. More than that, CTX could not only be used to monitor the early reduction of femoral neck bone mass and bone strength, and also be helpful to find patients with femoral neck osteopenia.

This study showed the cut-off value of serum BTMs for femoral neck osteopenia and OP. However, it is difficult to compare these results with previous studies because serum BTMs levels are dependent on the detection methods [30, 31]. Hu et al. [32] used electrochemiluminescence immunoassay to measure serum CTX levels and showed that the cut-off values of serum CTX, PINP, OC levels for diagnosing male OP were 0.38 ng/mL, 42.43 ng/mL, and 16.57 ng/mL, respectively. The cut-off values for diagnosis of female OP were 0.21 ng/mL, 32.90 ng/mL, and 13.90 ng/mL, respectively. Therefore, different detection methods can lead to differences in the level of BTMs and the cut-off value for the diagnosis of osteopenia and OP needs to be further investigated. In addition, we found that the AUC of CTX and OPN for femoral neck osteopenia was significantly higher than that of PINP and OC, the AUC of OPN and COL-I for femoral neck OP was significantly higher than that of PINP and OC (*P* < 0.05), suggesting that CTX and OPN may have higher diagnostic value in femoral neck osteopenia, OPN and COL-I may have higher diagnostic value in femoral neck OP.

The results of the above studies confirmed that CTX, OPN and COL-I all can well reflect the early change of femoral neck bone mass and bone strength, and help to early identify patients at high risk for the femoral neck osteopenia and OP. However, we should extract the femoral neck bone tissue from patients in the clinical work, it would not only not only increase patient suffering but also limit clinical use because the detection process is tedious, subject to error and time-consuming, although OPN and COL-I both can directly reflect reduced femoral neck bone mass and bone strength. In our study, we found that CTX had the strongest relationship with femoral neck BMD and L_max_ compared with OPN and COL-I, suggesting that CTX can better reflect the decline of femoral neck bone mass and bone strength. Furthermore, we found that CTX had significantly correlated with OPN and COL-I, indicating that CTX could be considered a surrogate marker for OPN and COL-I. We can be aware of the early change of femoral neck bone mass and bone strength from patients by serum CTX testing rather than removing femoral bone tissue.

Despite the significance of our findings, our study still has some limitations that are worthy of mention. We plan to enlarge the sample size in the following study and standardize the examination process of each indicator to obtain more reliable achievements and to guide clinical practice more precisely in further. Besides, our study didn’t compare BTMs to FRAX in classifying the groups or fracture status, and lack of compositional analysis (crosslinks, crystallinity, degree of mineralization) of the femoral neck samples. Therefore, these problems will be improved in later research.

## Conclusions

In summary, compared with other indicators, serum CTX was more sensitive to differences in bone mass and bone strength of femoral neck, and could be considered as a surrogate marker for OPN and COL-I. Therefore, early measurement of serum CTX not only help us understand early changes of femoral neck bone mass and bone strength more intuitively and efficiently, but also facilitate the diagnosis of osteopenia and provide a theoretical basis for delaying the occurrence of femoral neck OP and fragility fractures.

## Supplementary Information

**Additional file 1. **

**Additional file 2. **

## Data Availability

The datasets used and analyzed during the current study are available from the corresponding author Da Liu on reasonable request.

## Abbreviations

ANOVA: Analysis of variance
AUC: The area under the curve
BMD: Bone mineral density
BMI: Body mass index
BTMs: Bone turnover markers
COL-I: Type I collagen
CTX: C-terminal telopeptide of type I collagen
DXA: Dual energy X-ray absorptiometry
ELISA: Enzyme-linked immunosorbent assay
OC: Osteocalcin
OP: Osteoporosis
OPN: Osteopontin
PBS: Phosphate-buffered saline
PINP: Type I procollagen N-terminal propeptide
PVDF: Polyvinylidene fluoride
ROC: Receiver operating characteristic curve
