# Supplementary material for: Association of bone-related biomarkers with femoral neck bone strength
Source: BMC Musculoskelet Disord. 2022 May 21;23:482. doi: 10.1186/s12891-022-05427-1 (PMC9123746; doi:10.1186/s12891-022-05427-1)
Supplement: Supplementary file 1 — Additional file 1. [file 12891_2022_5427_MOESM1_ESM.pdf]

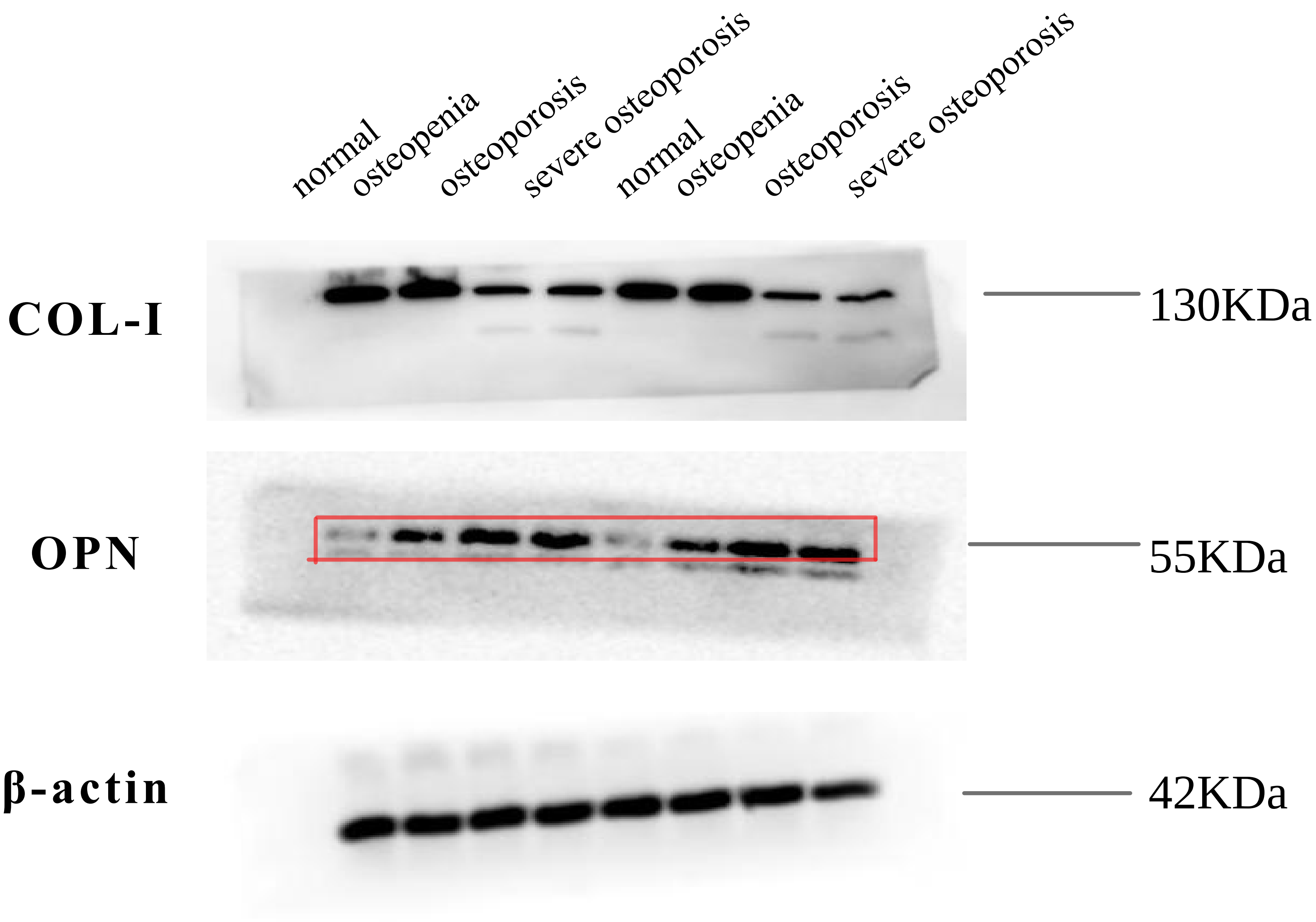
normal
osteopenia
osteoporosis
severe osteoporosis
normal
osteopenia
osteoporosis
severe osteoporosis
COL-I
OPN
β-actin
130KDa
55KDa
42KDa

Protein expression levels in OPN and COL-I were determined via western blot analysis. The bands on the membrane were visualized and analyzed using UVP Bioimaging Systems (UVP, LLC, Phoenix, AZ, USA), and β﹣actin was used as a loading control. Prior to hybridisation with primary antibodies, membranes were cut at the each expected blots point. For all western blots shown, femoral neck samples were derived from the same experiment and processed in parallel.

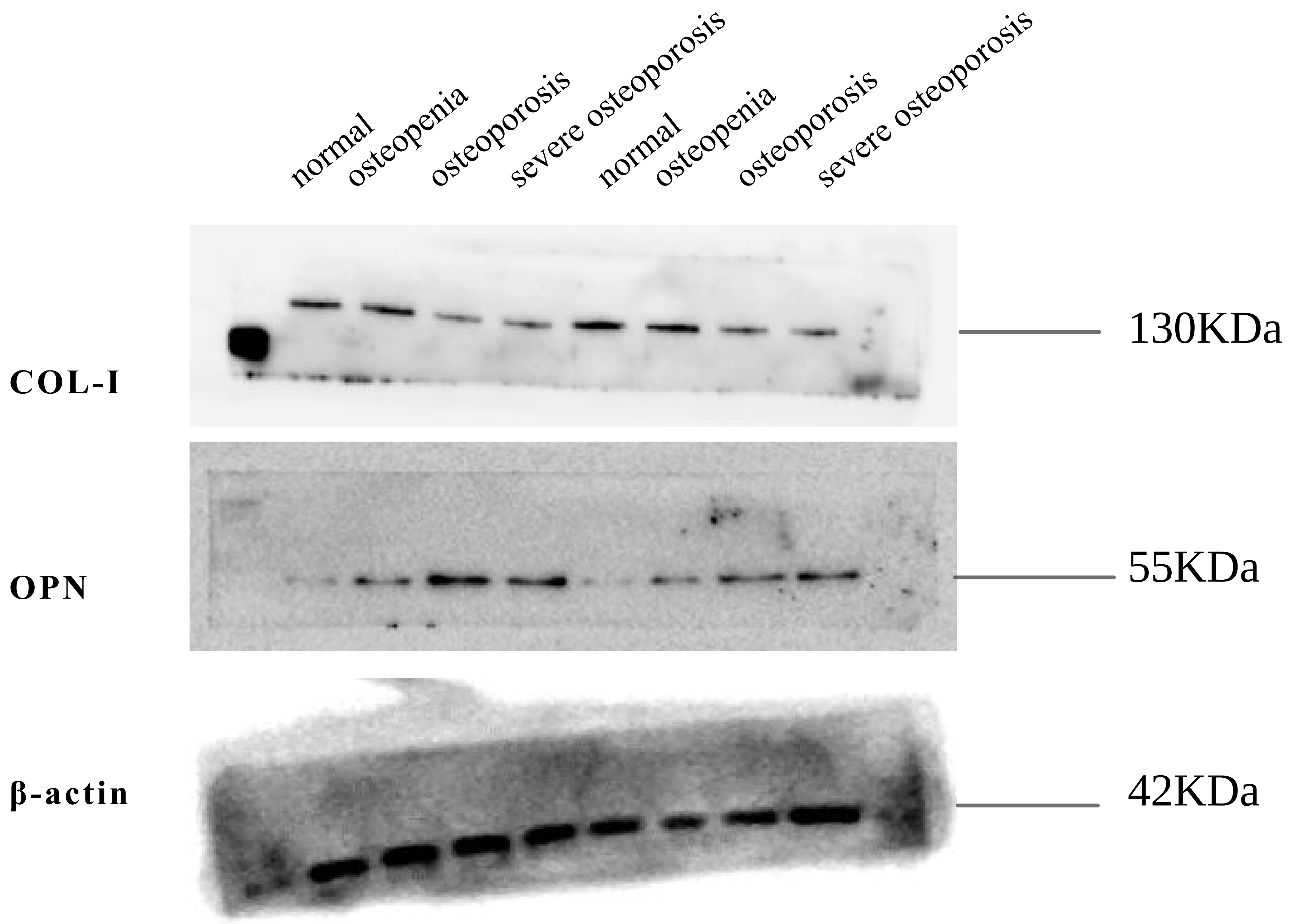
normal
osteopenia
osteoporosis
severe osteoporosis
normal
osteopenia
osteoporosis
severe osteoporosis
COL-I
OPN
β-actin
130KDa
55KDa
42KDa

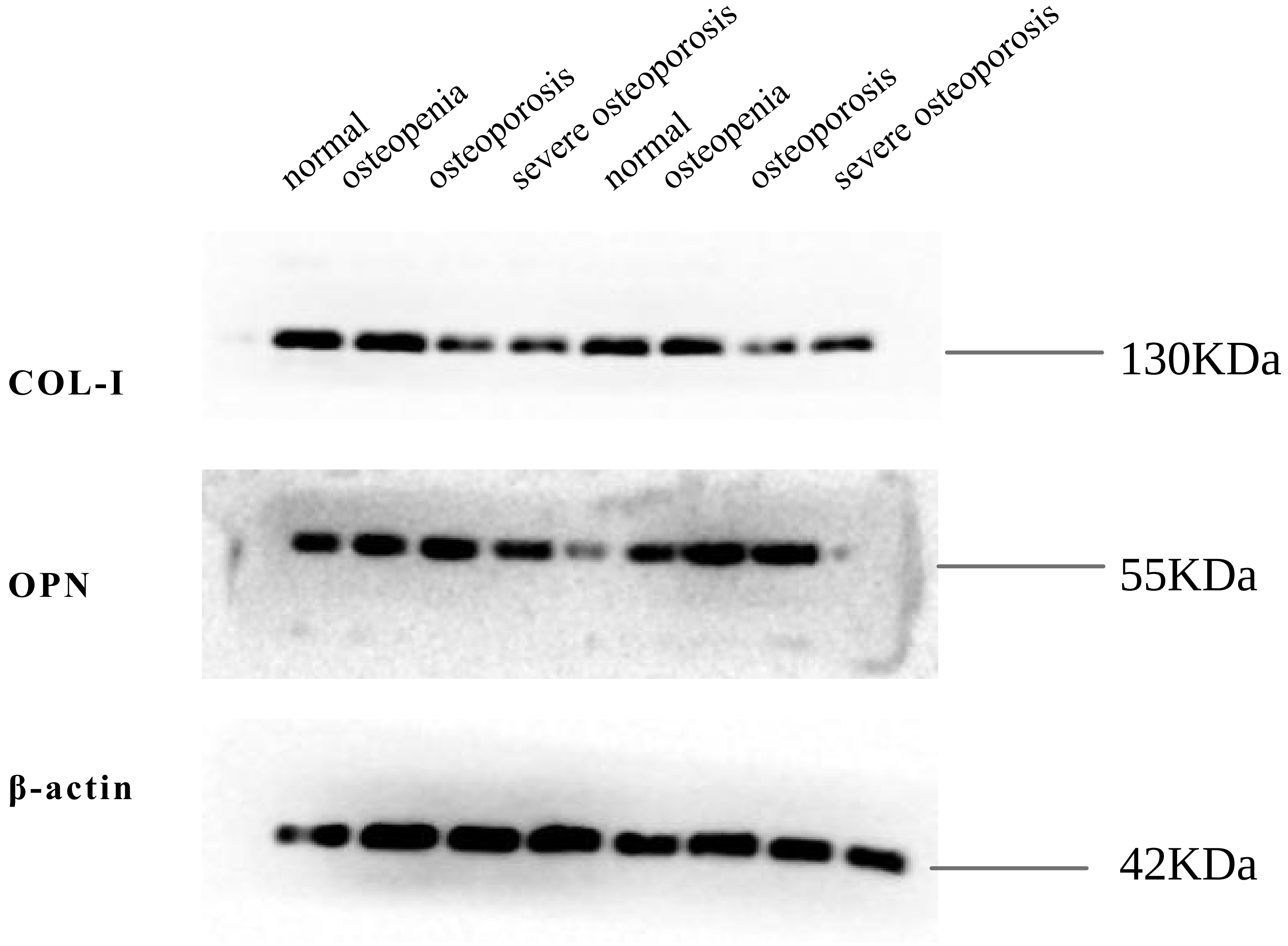
normal
osteopenia
osteoporosis
severe osteoporosis
normal
osteopenia
osteoporosis
severe osteoporosis
COL-I
130KDa
OPN
55KDa
β-actin
42KDa

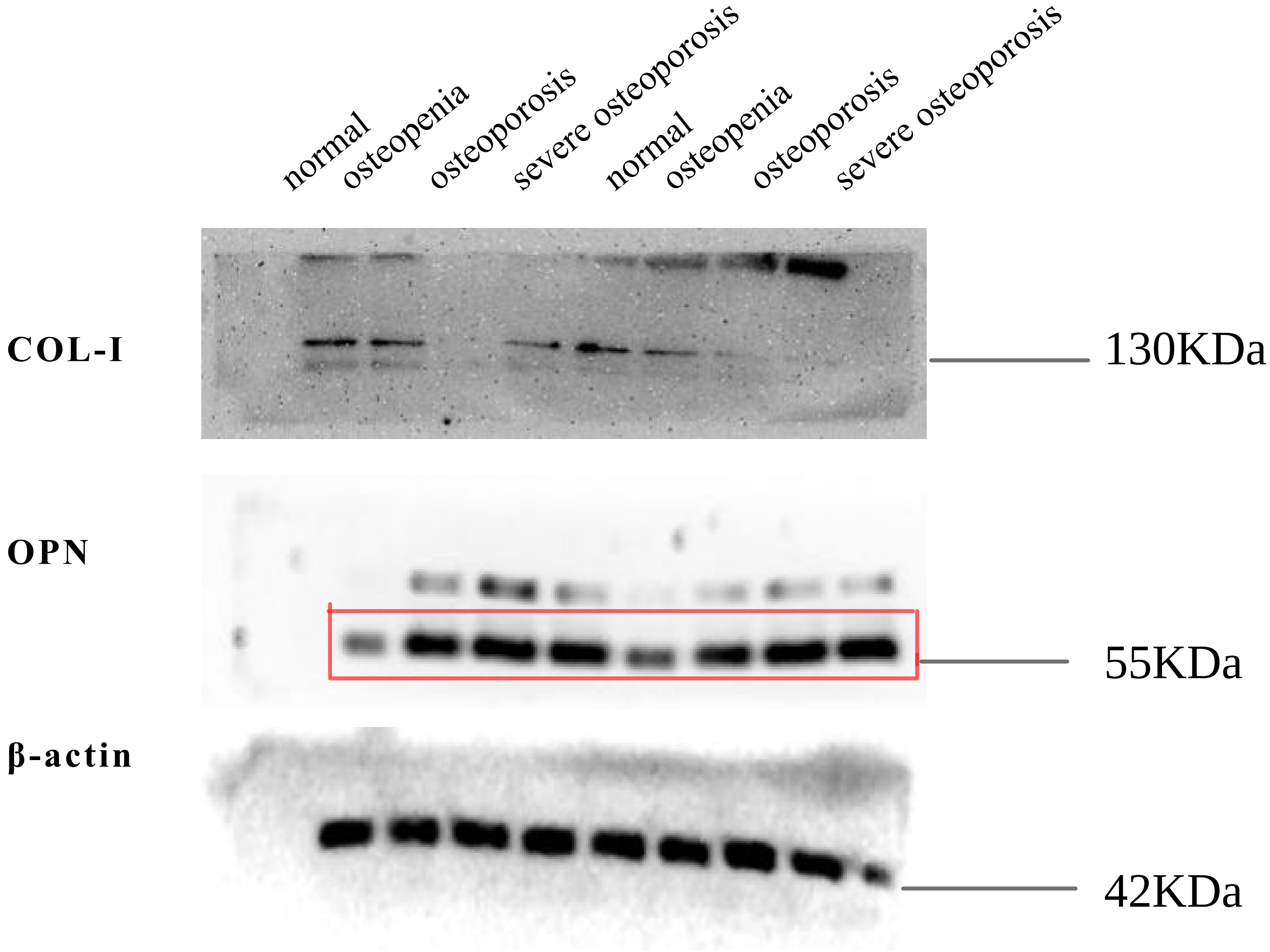
normal
osteopenia
osteoporosis
severe osteoporosis
normal
osteopenia
osteoporosis
severe osteoporosis
COL-I
130KDa
OPN
55KDa
β-actin
42KDa

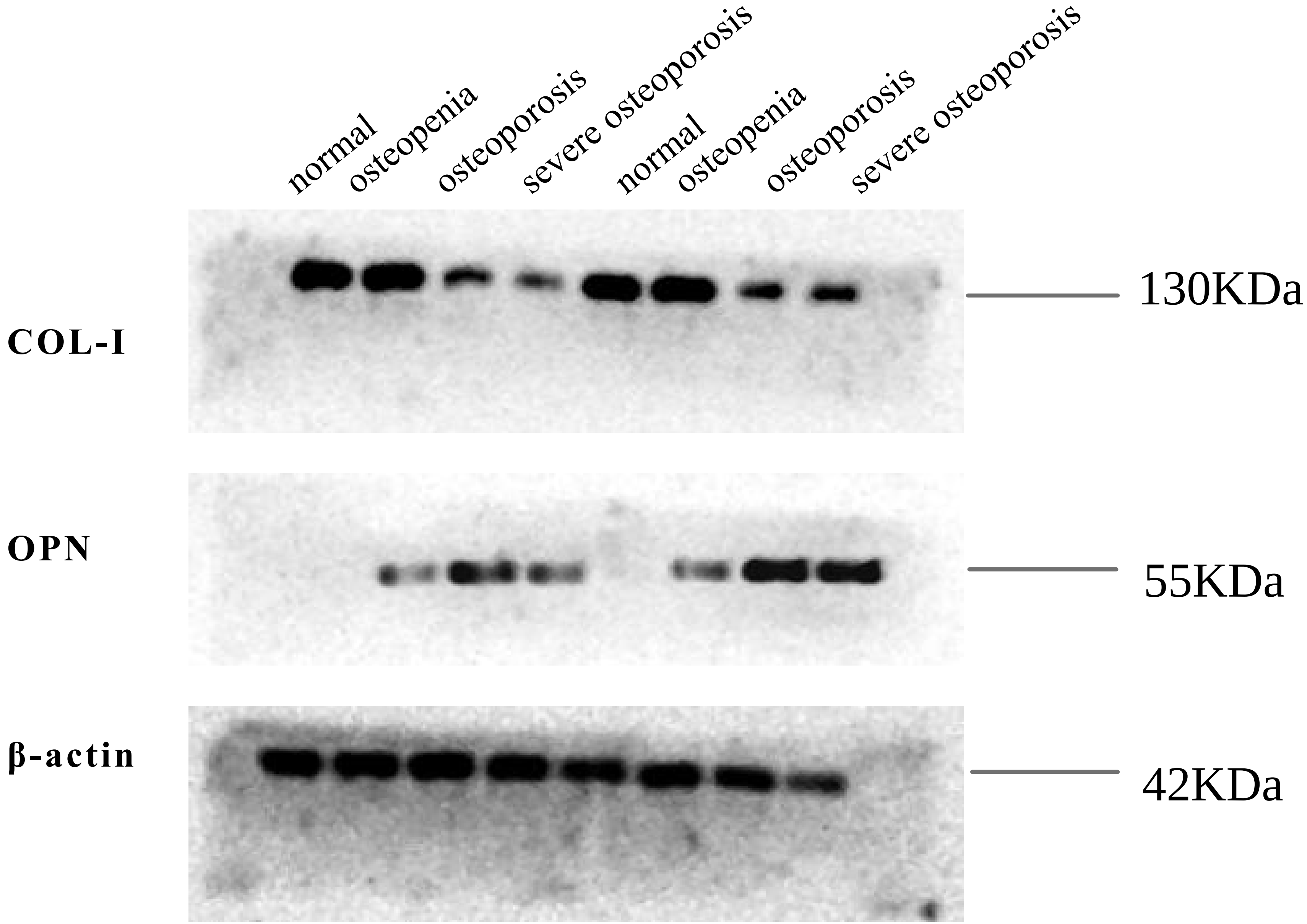
normal
osteopenia
osteoporosis
severe osteoporosis
normal
osteopenia
osteoporosis
severe osteoporosis
COL-I
OPN
β-actin
130KDa
55KDa
42KDa

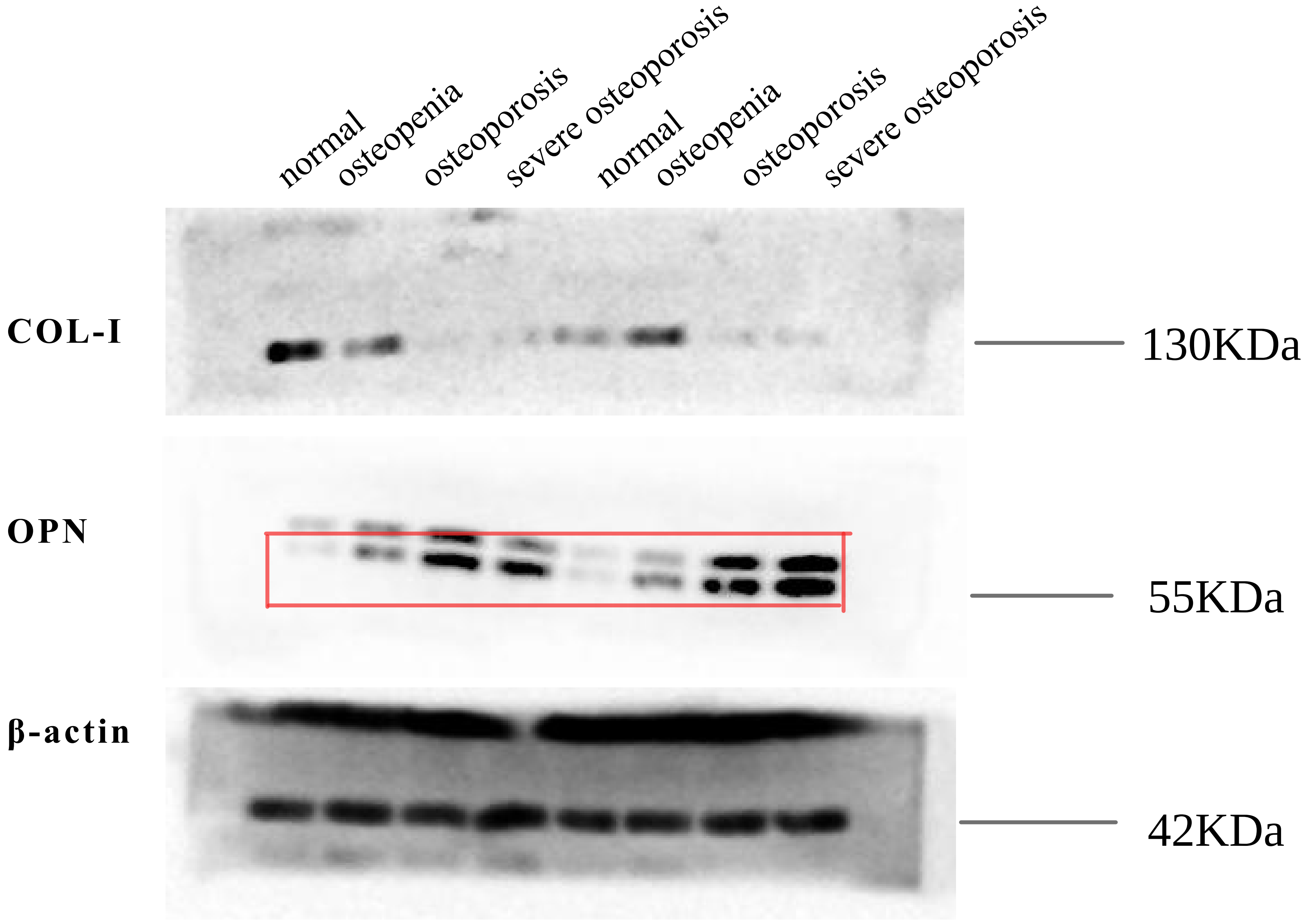
normal
osteopenia
osteoporosis
severe osteoporosis
normal
osteopenia
osteoporosis
severe osteoporosis
COL-I
130KDa
OPN
55KDa
β-actin
42KDa

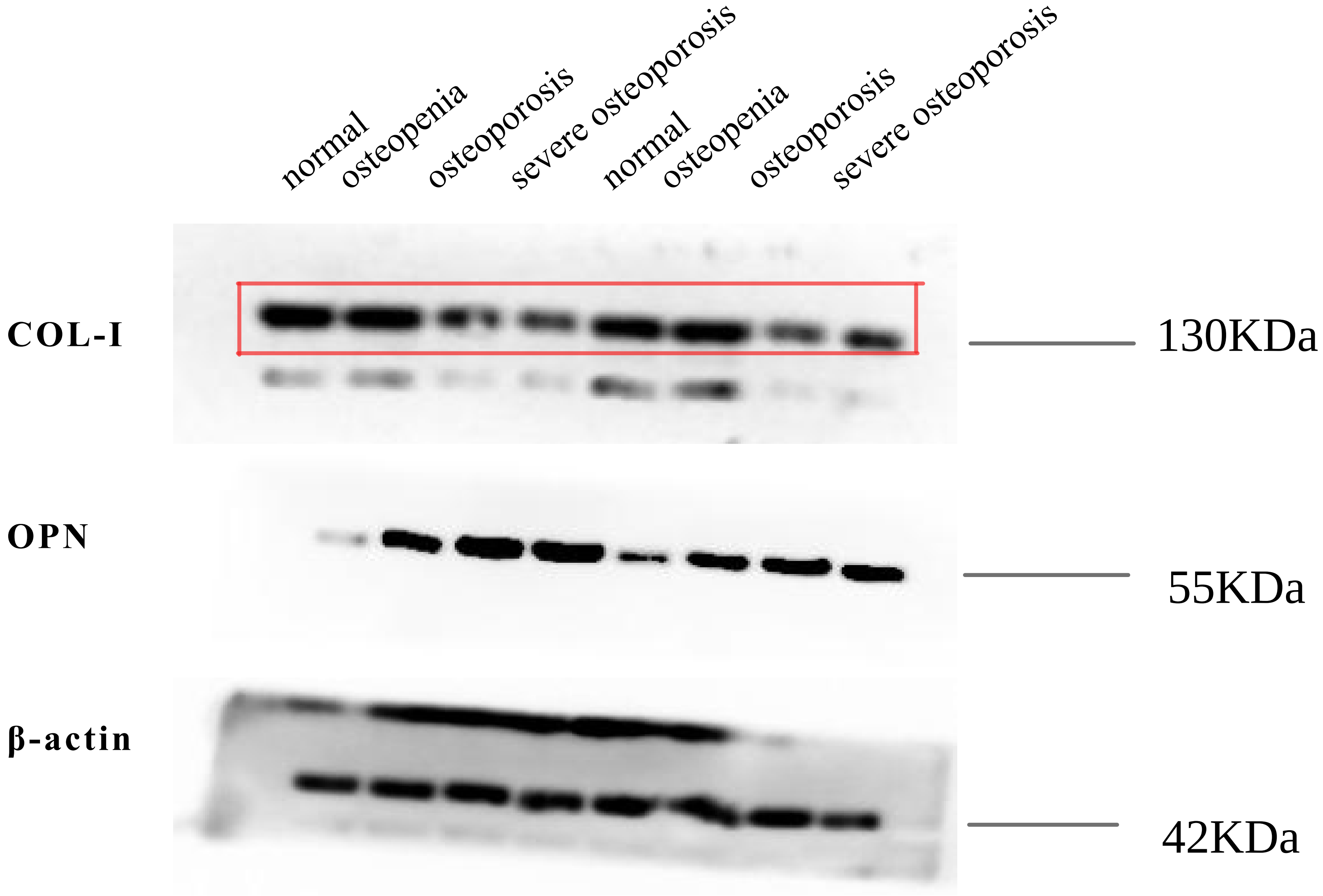
normal
osteopenia
osteoporosis
severe osteoporosis
normal
osteopenia
osteoporosis
severe osteoporosis
COL-I
OPN
β-actin
130KDa
55KDa
42KDa

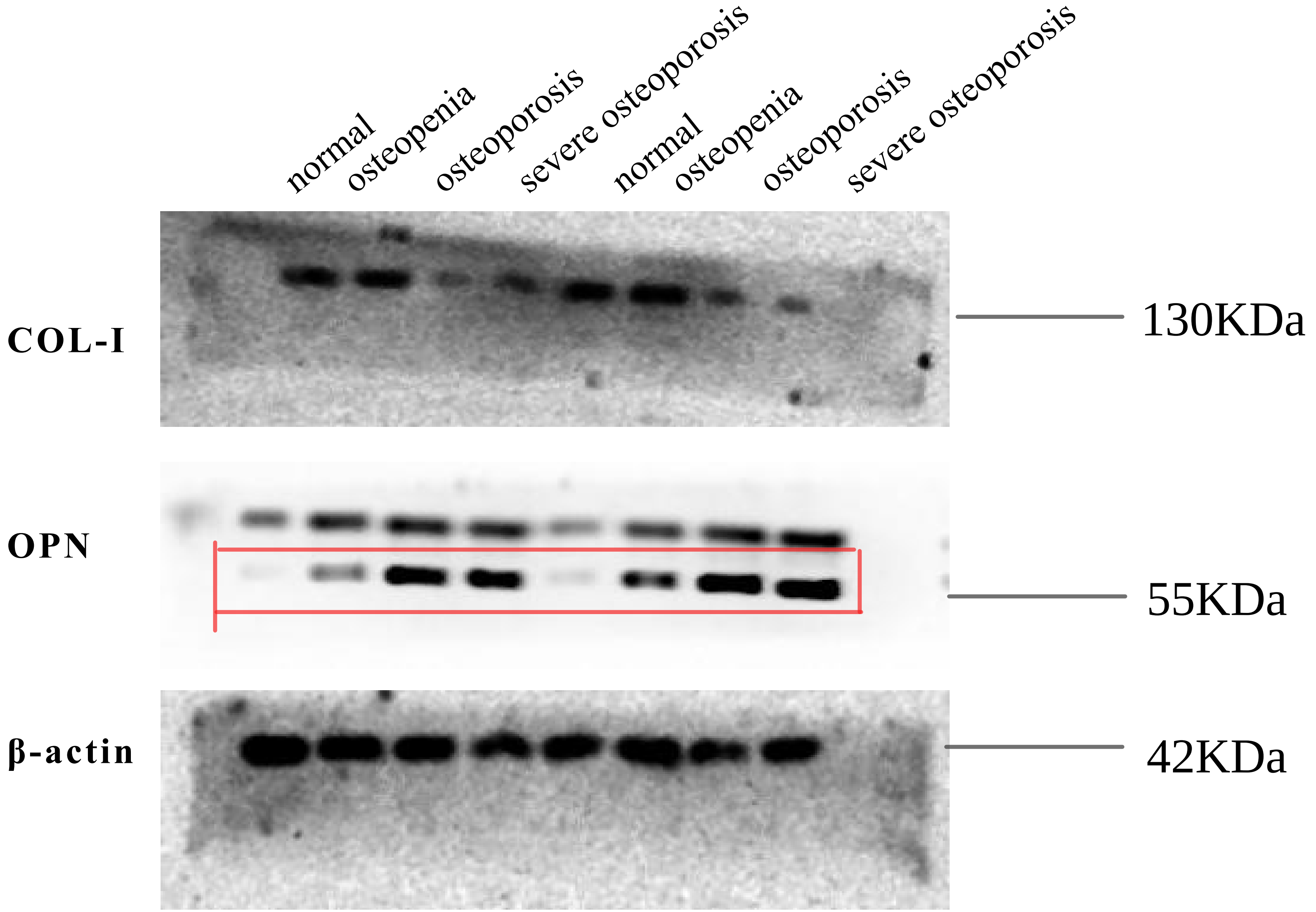
normal
osteopenia
osteoporosis
severe osteoporosis
normal
osteopenia
osteoporosis
severe osteoporosis
COL-I
130KDa
OPN
55KDa
β-actin
42KDa

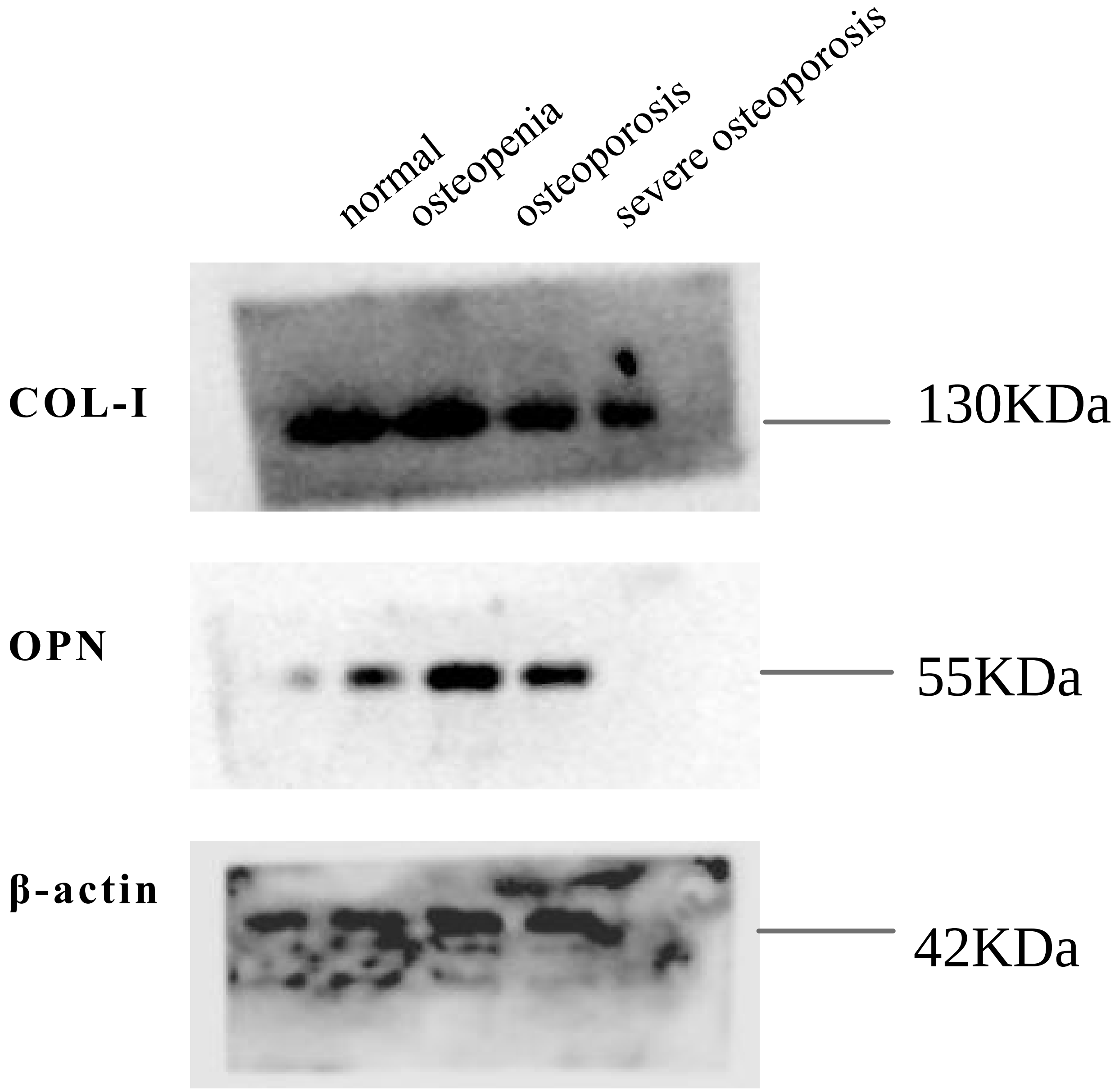
normal
osteopenia
osteoporosis
severe osteoporosis
COL-I
130KDa
OPN
55KDa
β-actin
42KDa

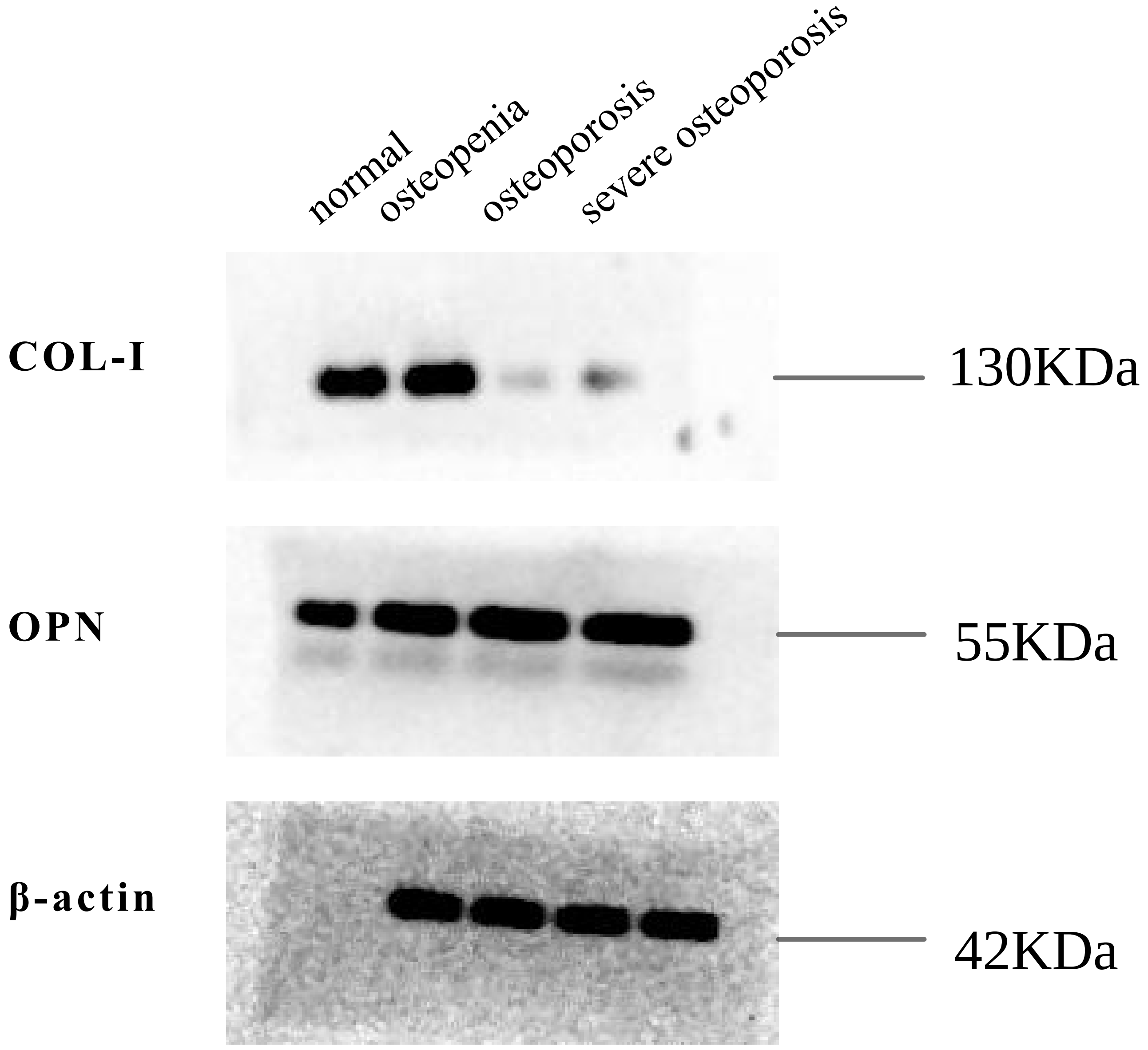
normal
osteopenia
osteoporosis
severe osteoporosis
COL-I
130KDa
OPN
55KDa
β-actin
42KDa

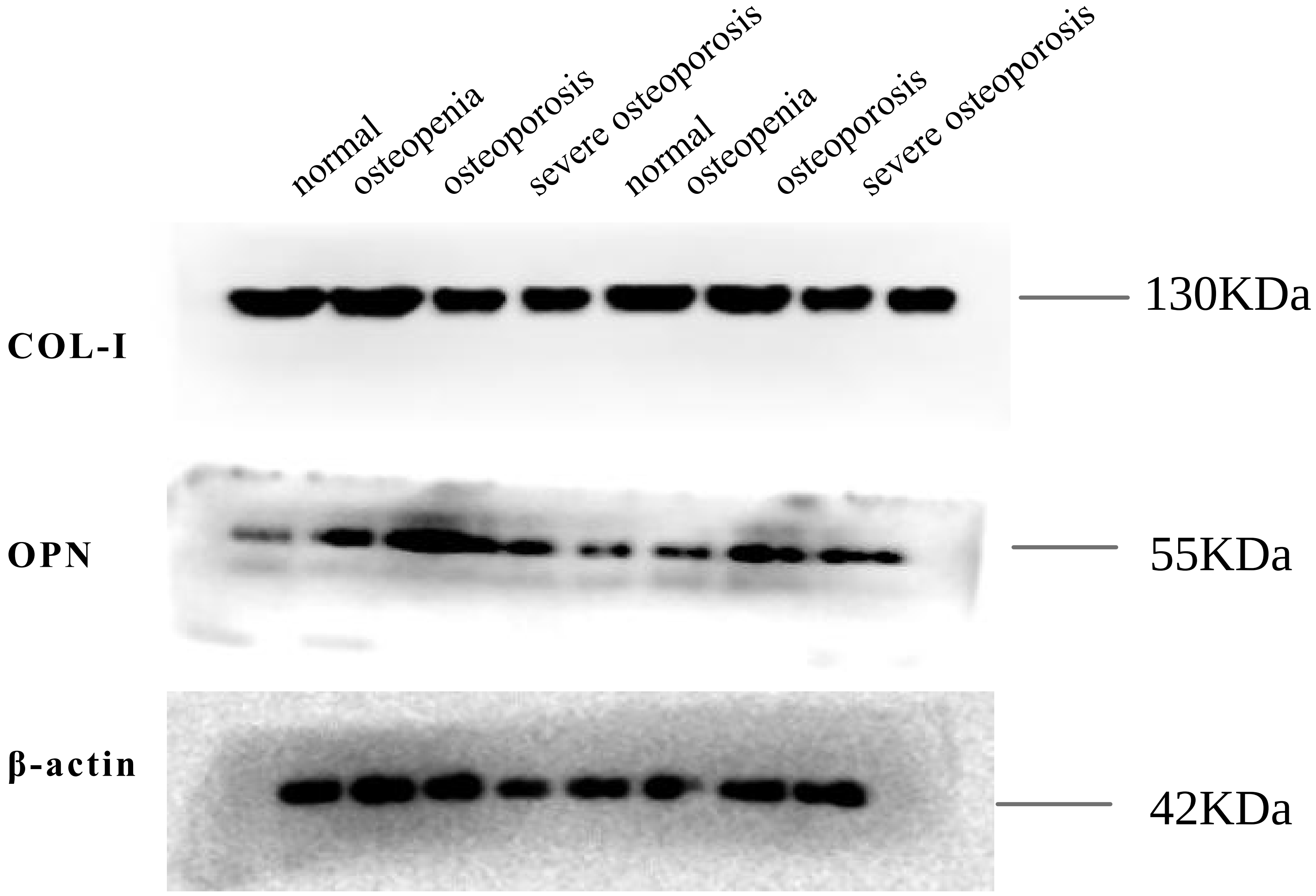
normal
osteopenia
osteoporosis
severe osteoporosis
normal
osteopenia
osteoporosis
severe osteoporosis
COL-I
OPN
β-actin
130KDa
55KDa
42KDa

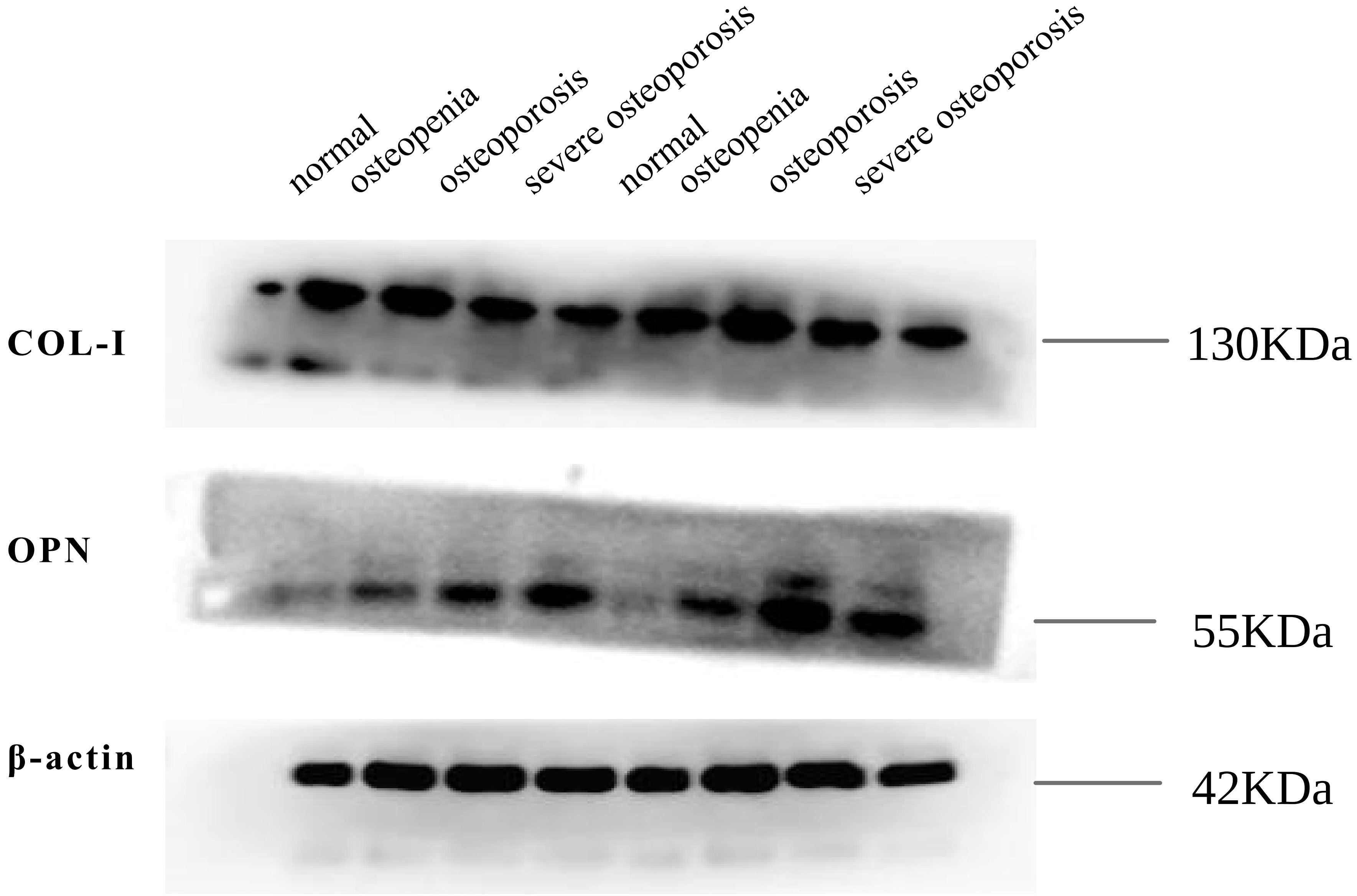
normal
osteopenia
osteoporosis
severe osteoporosis
normal
osteopenia
osteoporosis
severe osteoporosis
COL-I
130KDa
OPN
55KDa
β-actin
42KDa

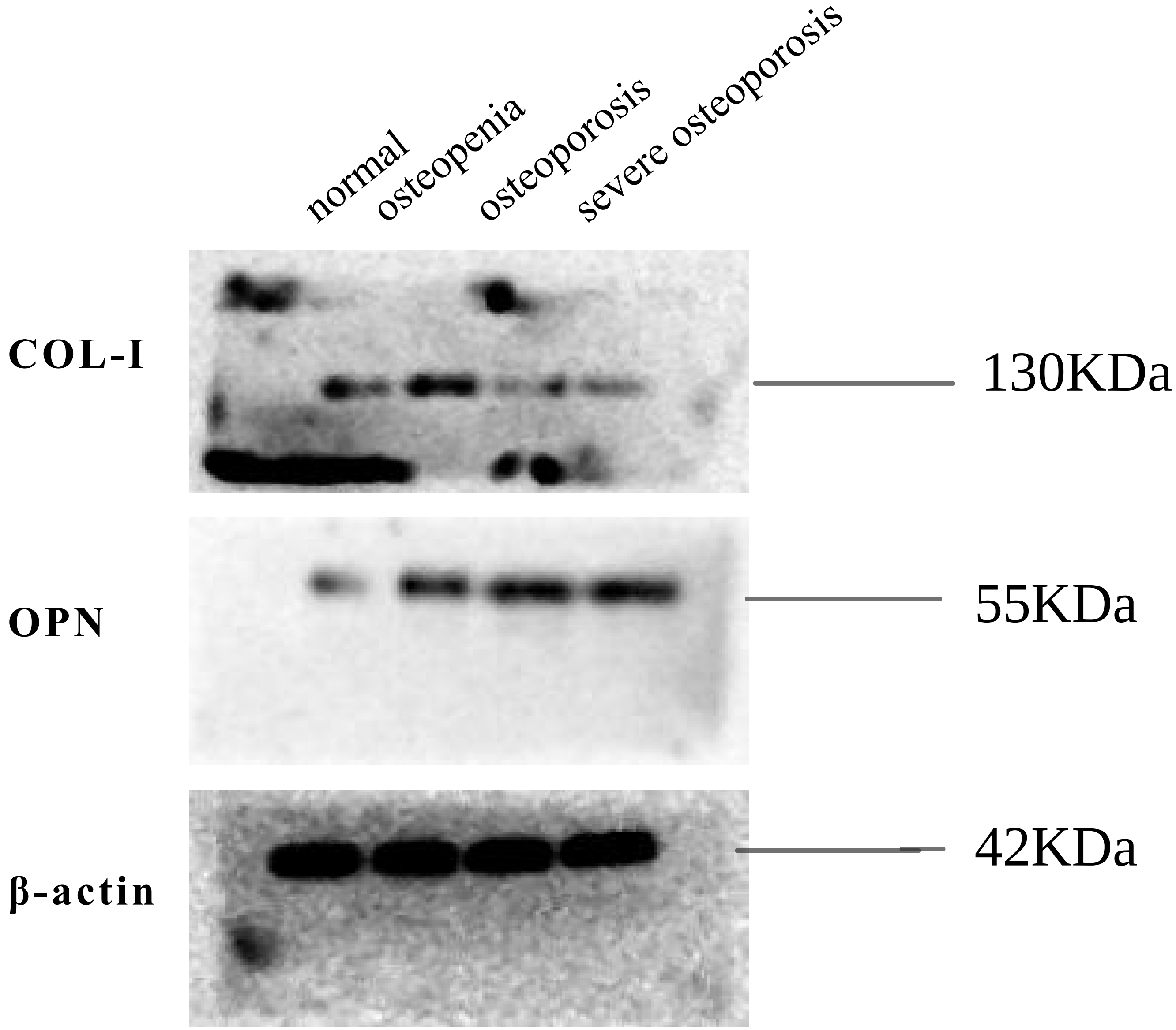
normal
osteopenia
osteoporosis
severe osteoporosis
COL-I
130KDa
OPN
55KDa
β-actin
42KDa

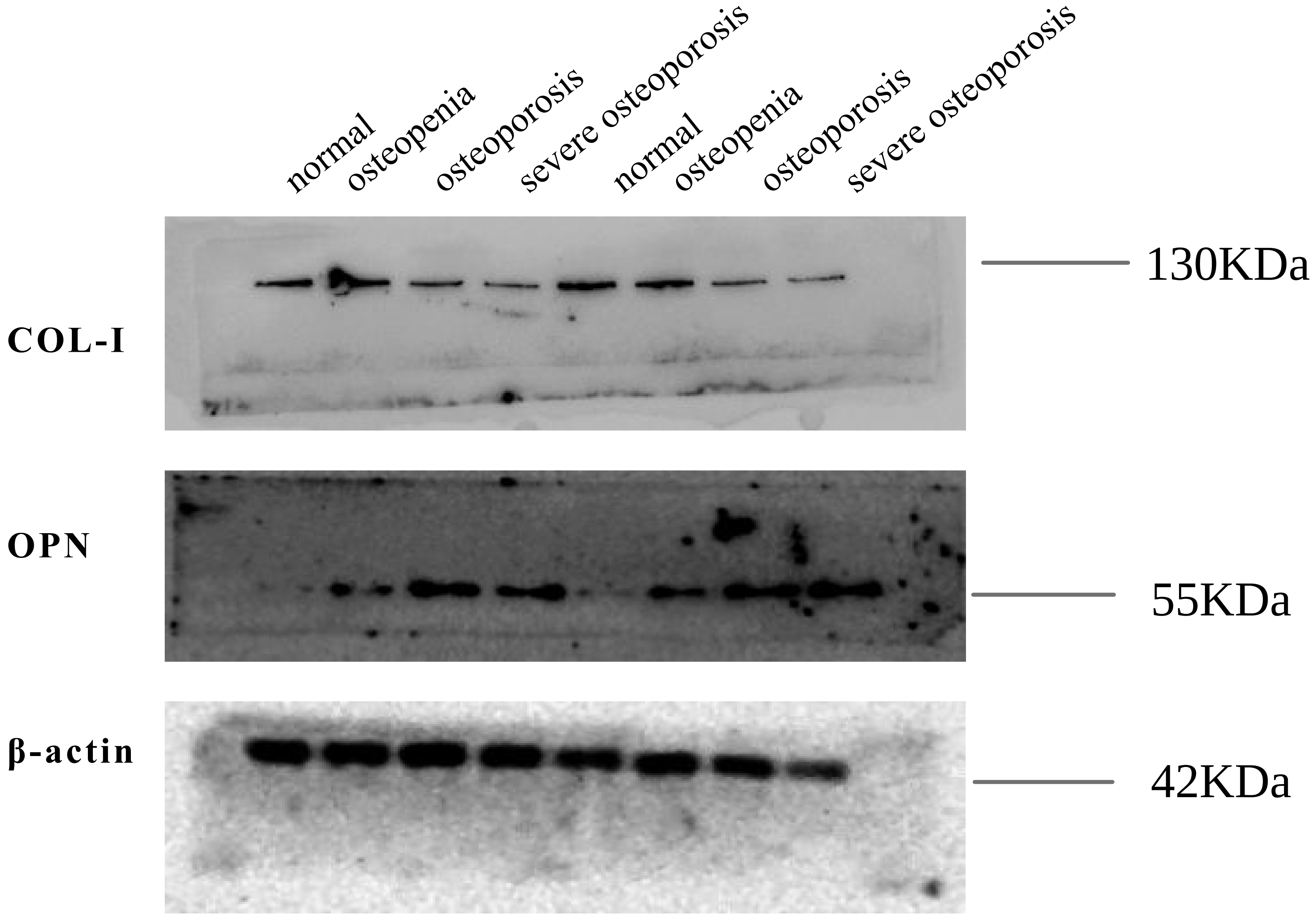
normal
osteopenia
osteoporosis
severe osteoporosis
normal
osteopenia
osteoporosis
severe osteoporosis
COL-I
OPN
β-actin
130KDa
55KDa
42KDa

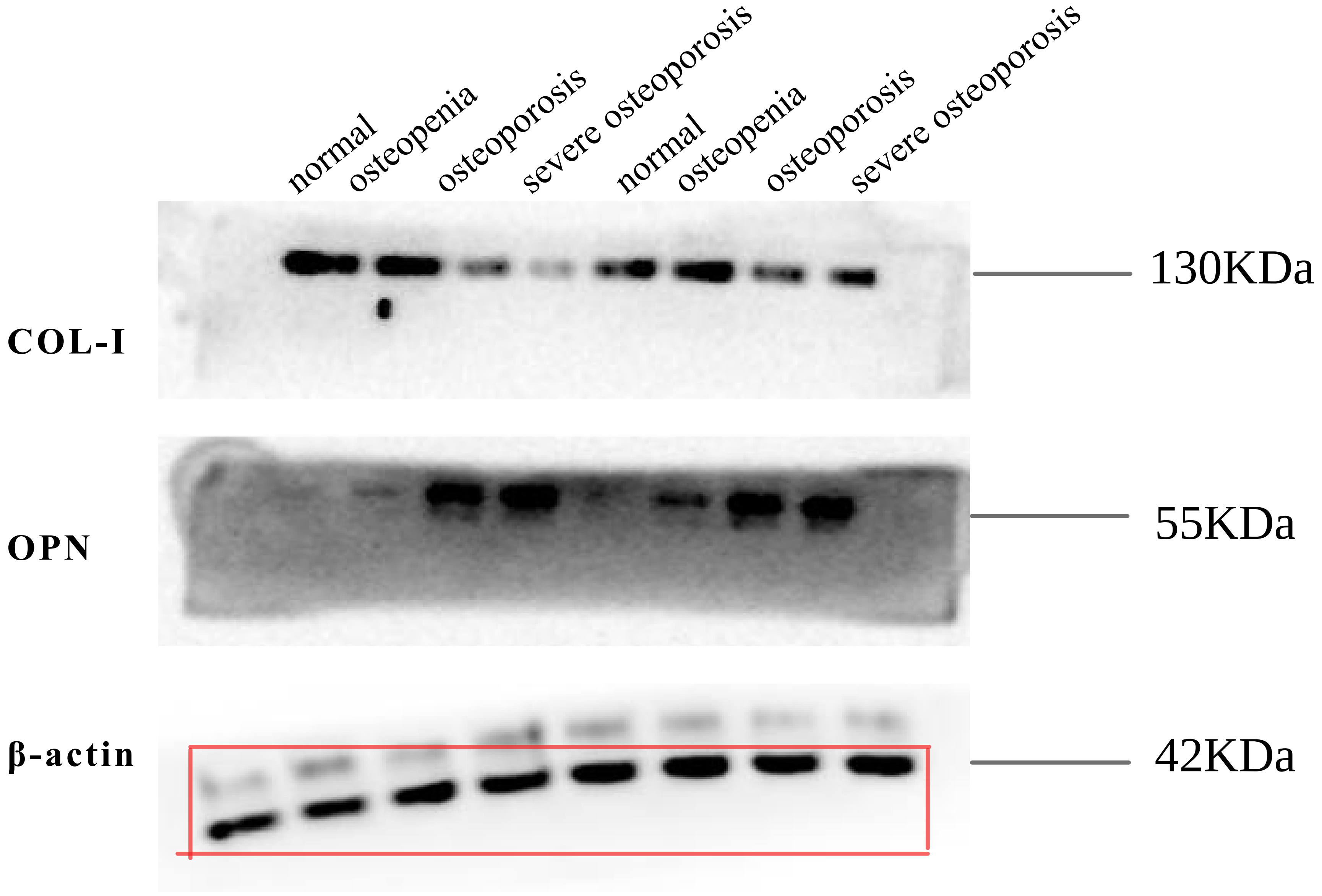
normal
osteopenia
osteoporosis
severe osteoporosis
normal
osteopenia
osteoporosis
severe osteoporosis
COL-I
OPN
β-actin
130KDa
55KDa
42KDa
