# Supplementary figures and images for: Association of bone-related biomarkers with femoral neck bone strength
Source: BMC Musculoskelet Disord. 2022 May 21;23:482. doi: 10.1186/s12891-022-05427-1 (PMC9123746; doi:10.1186/s12891-022-05427-1)

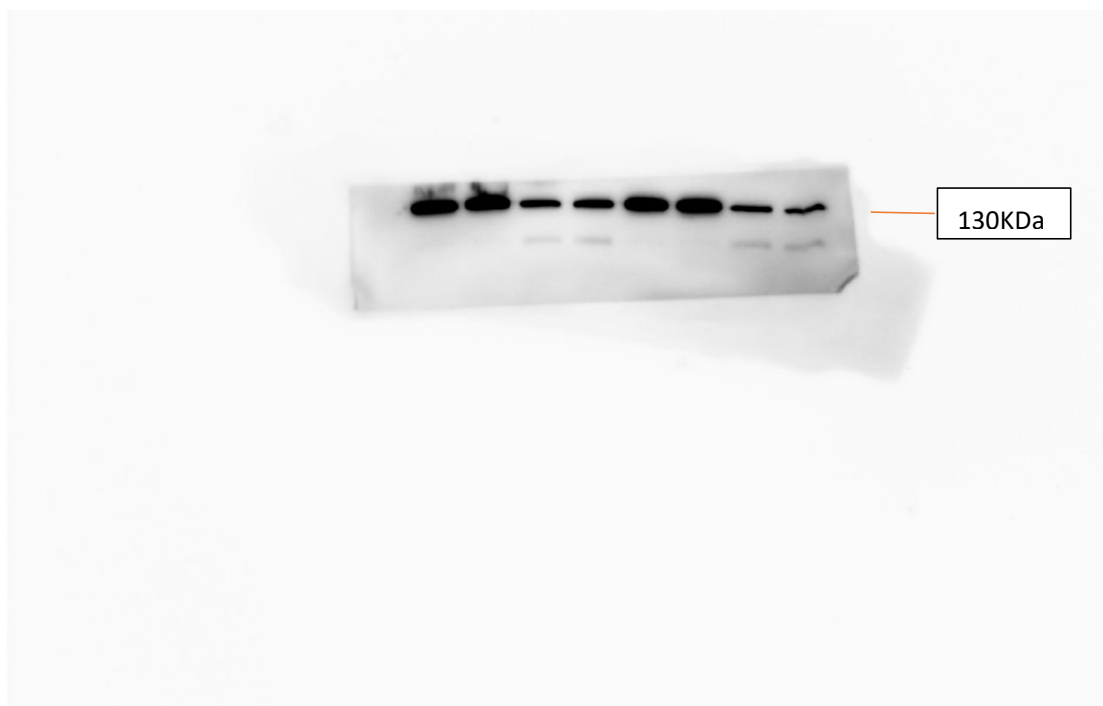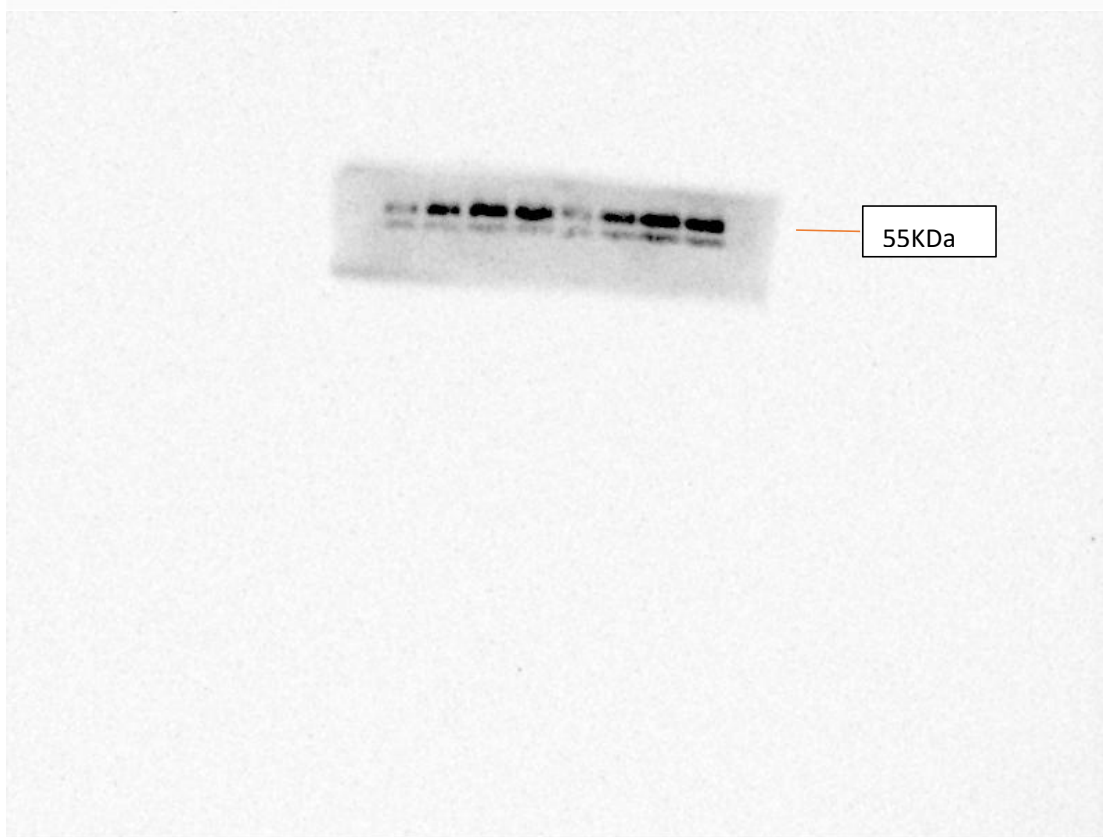

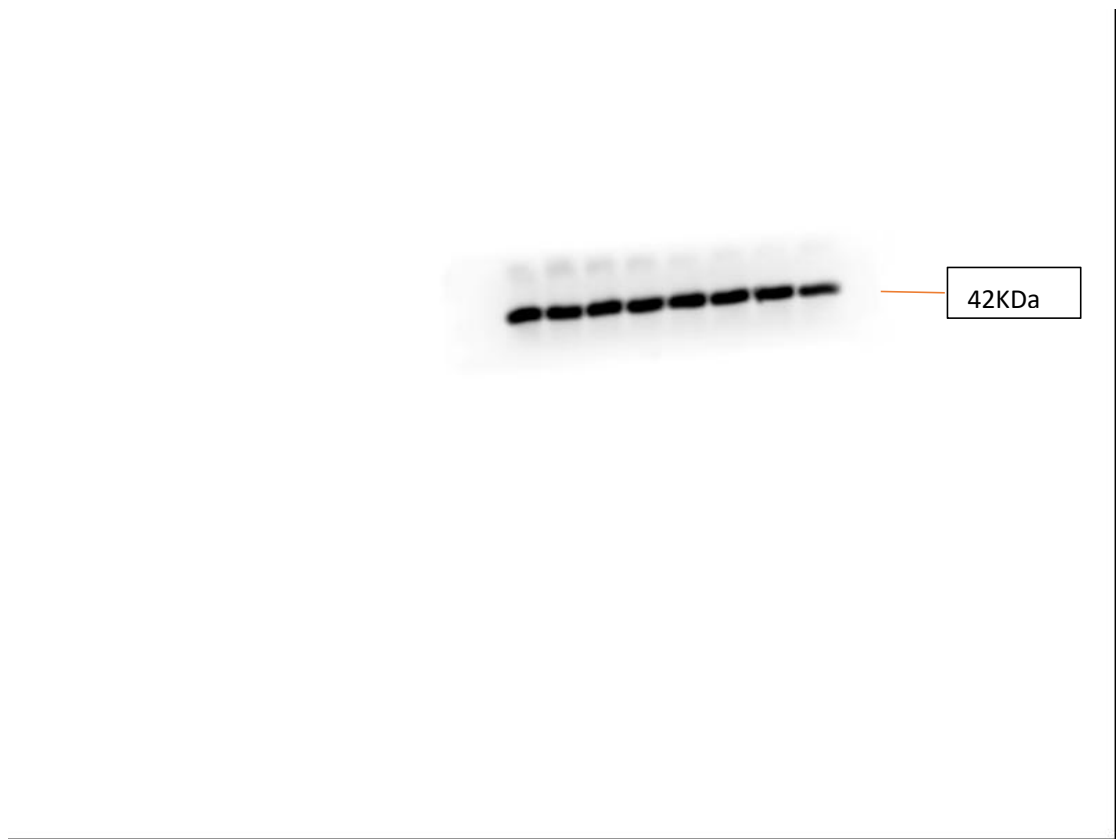

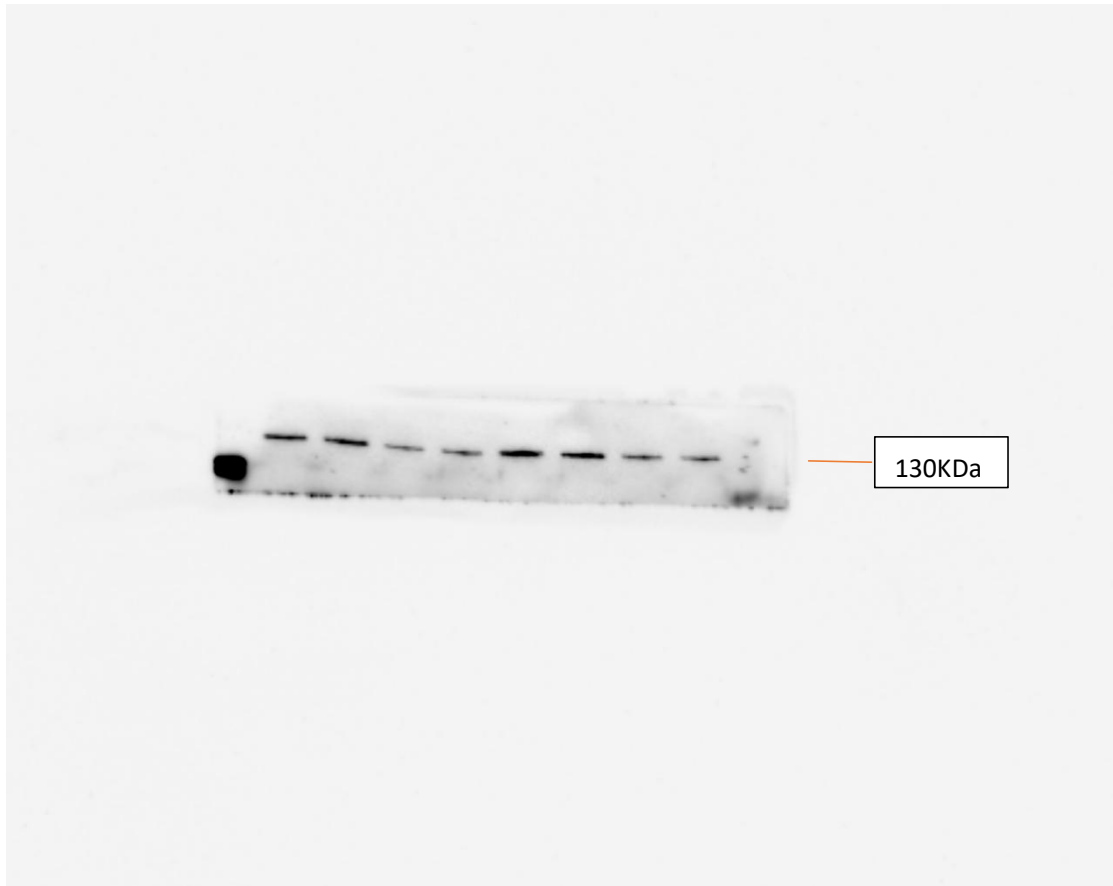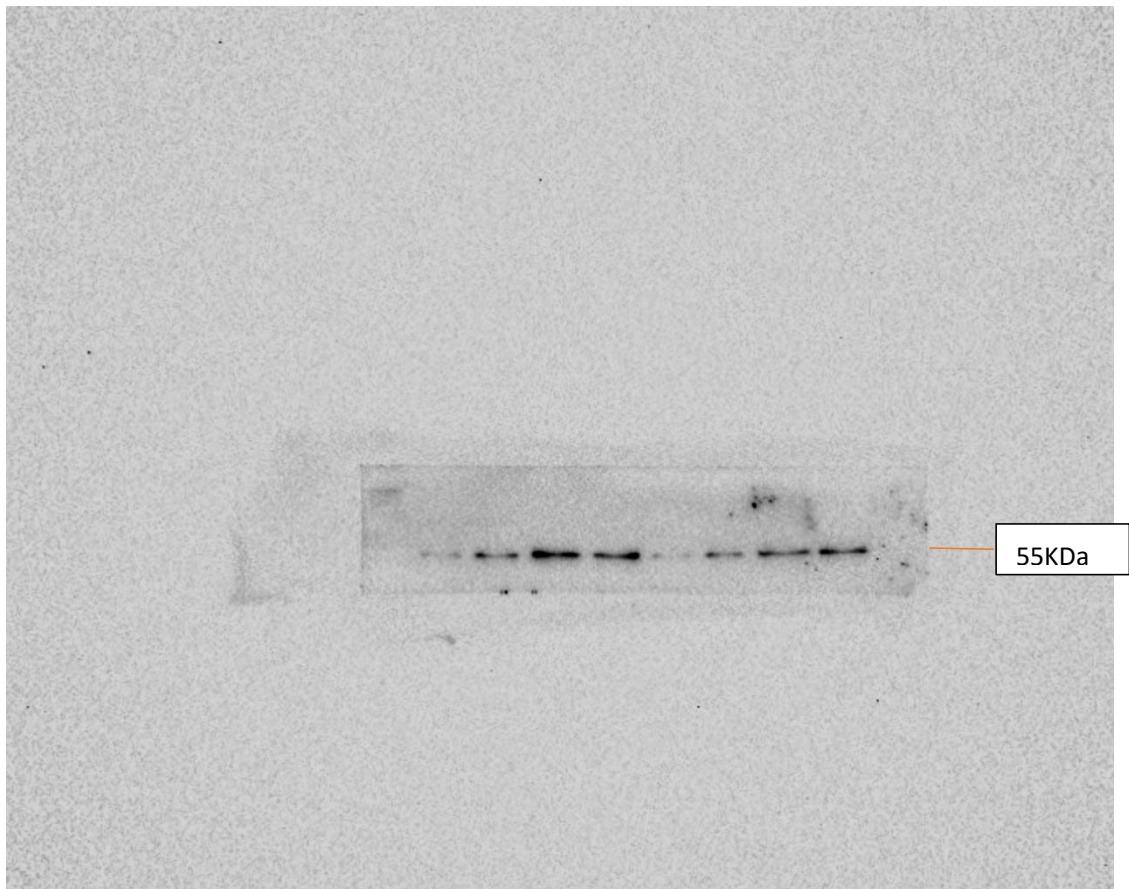

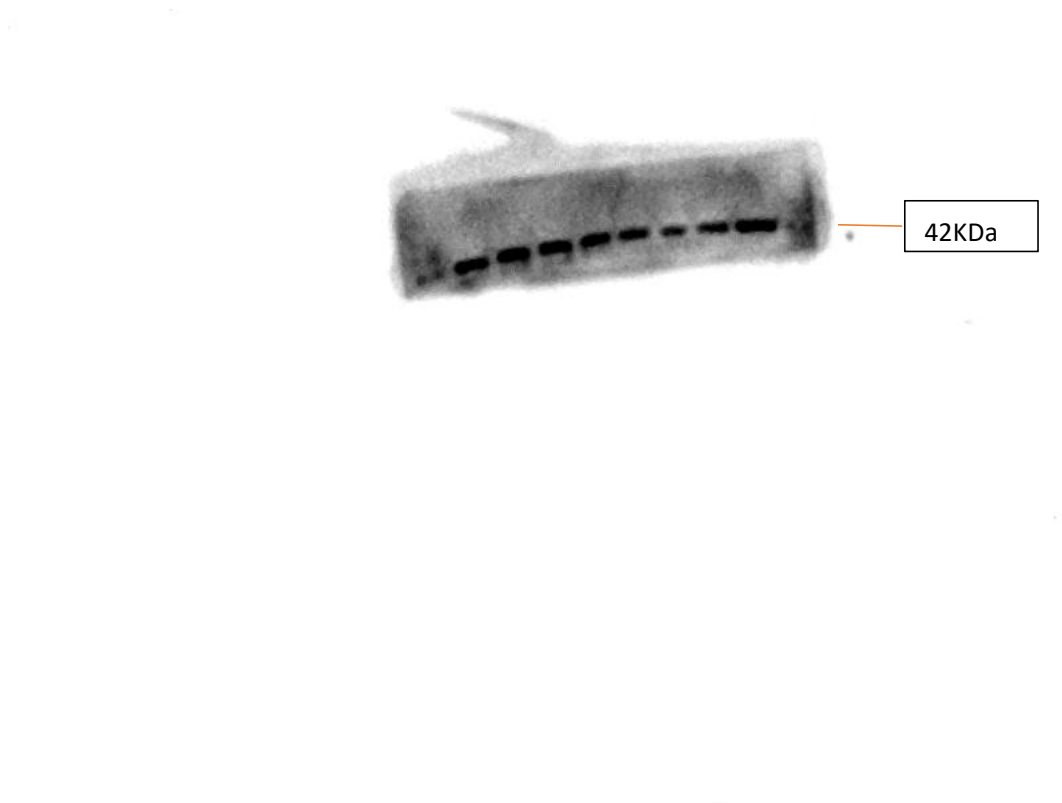

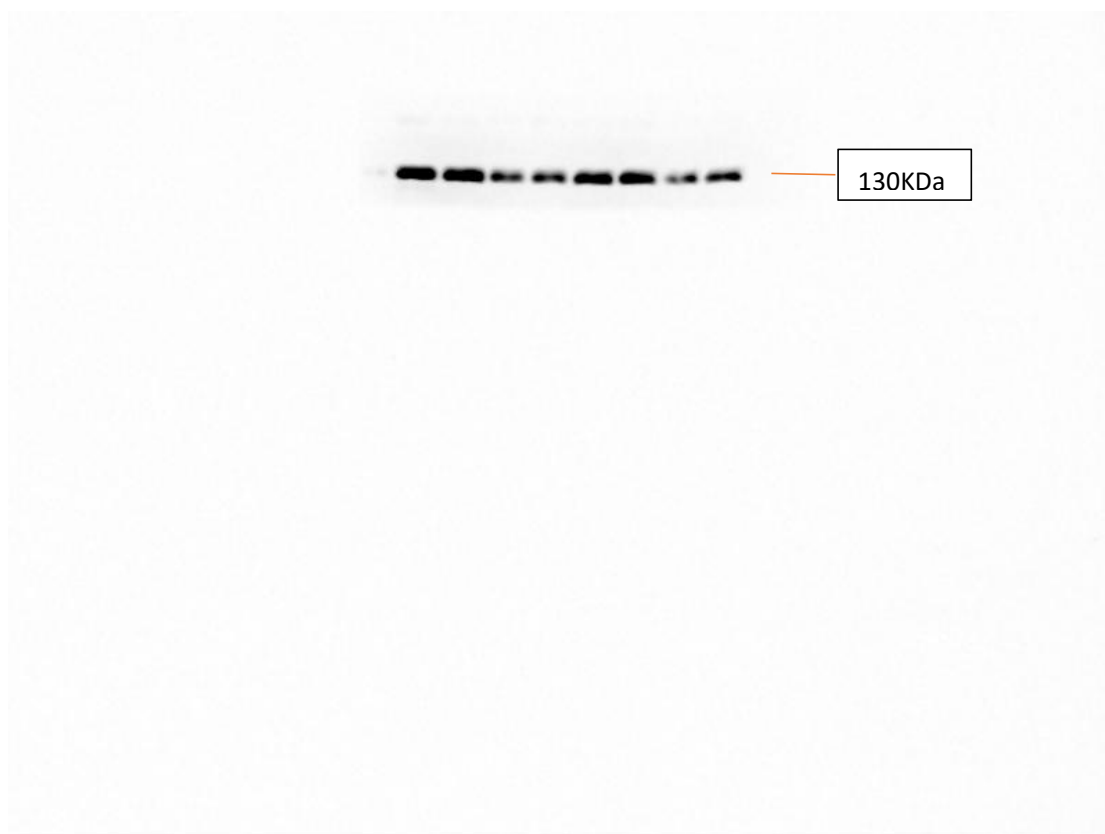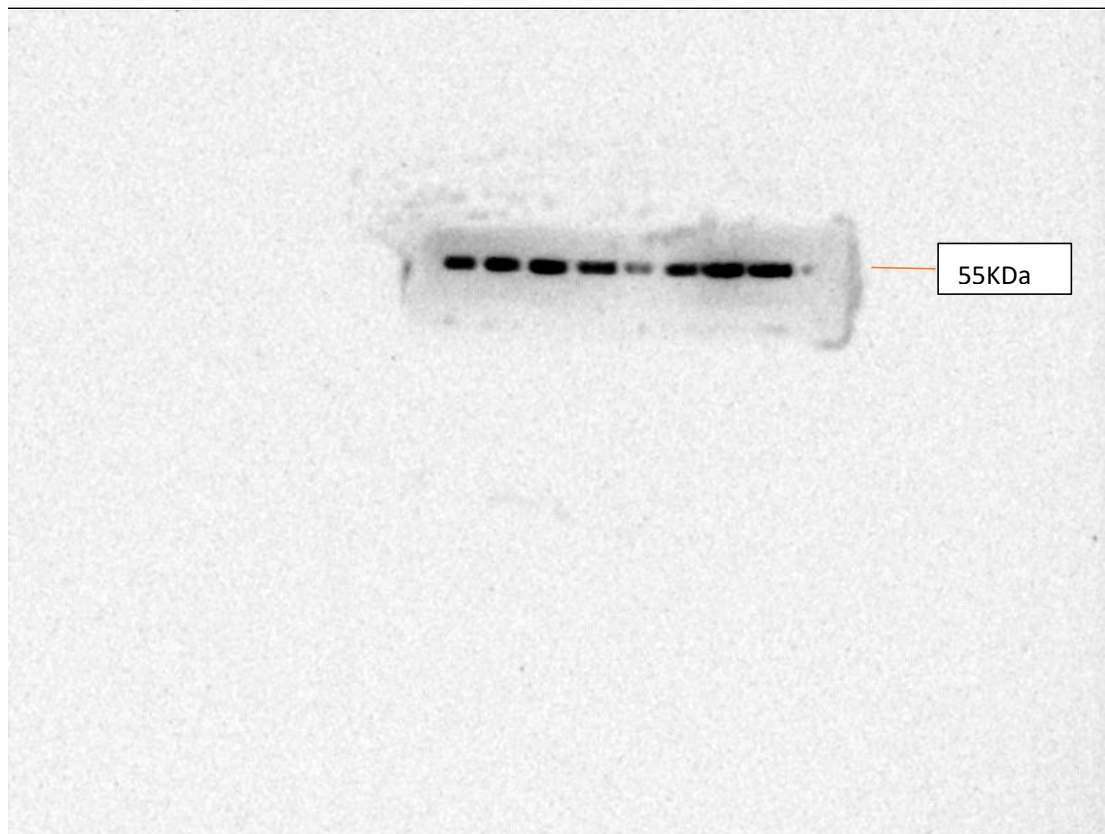

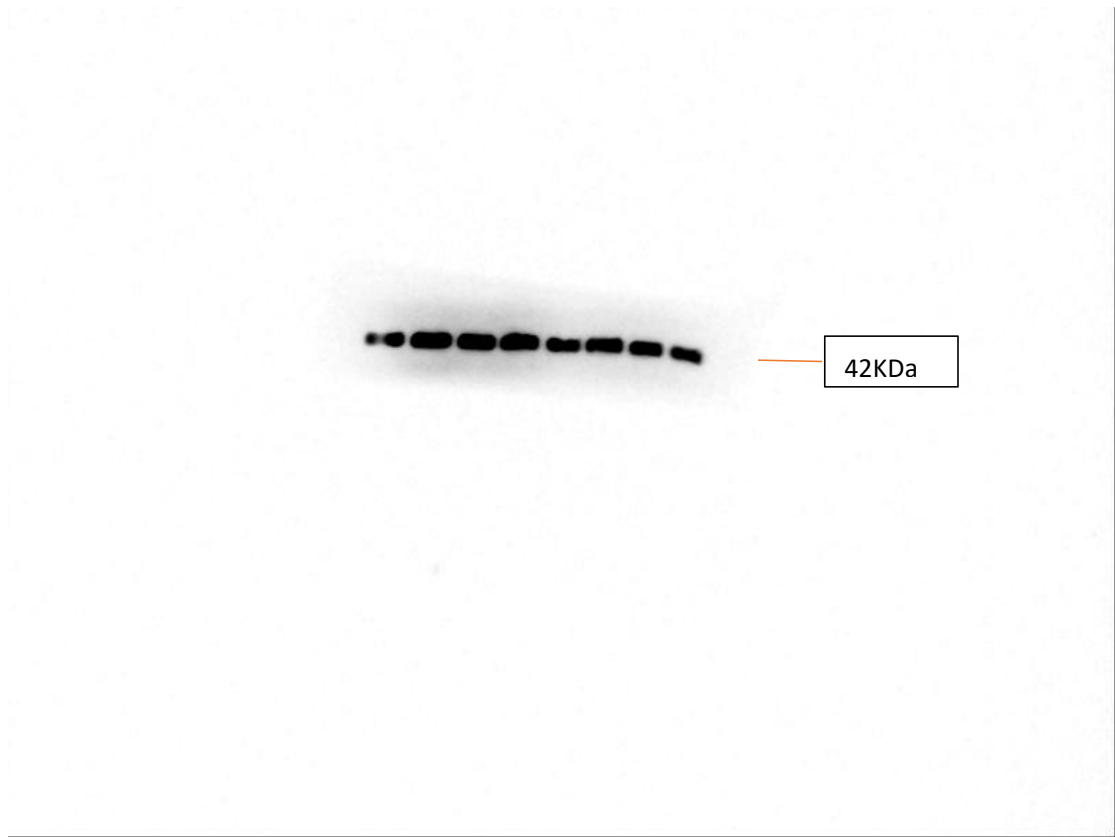

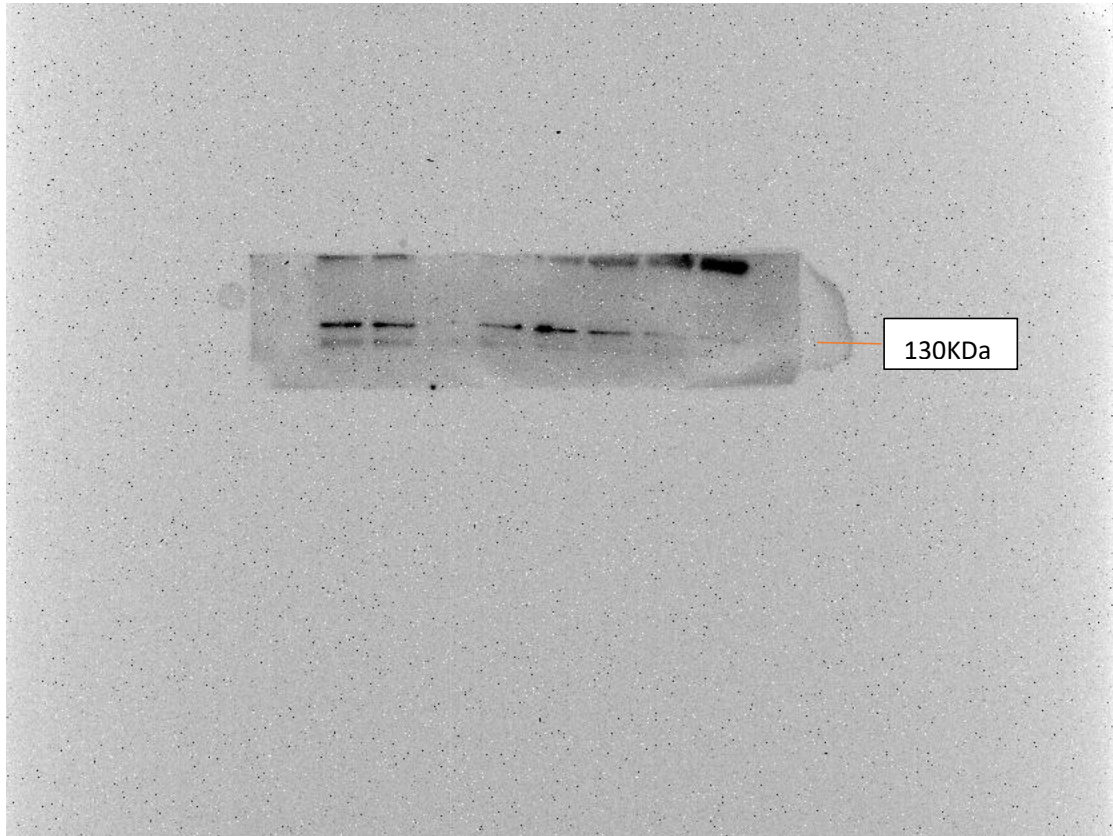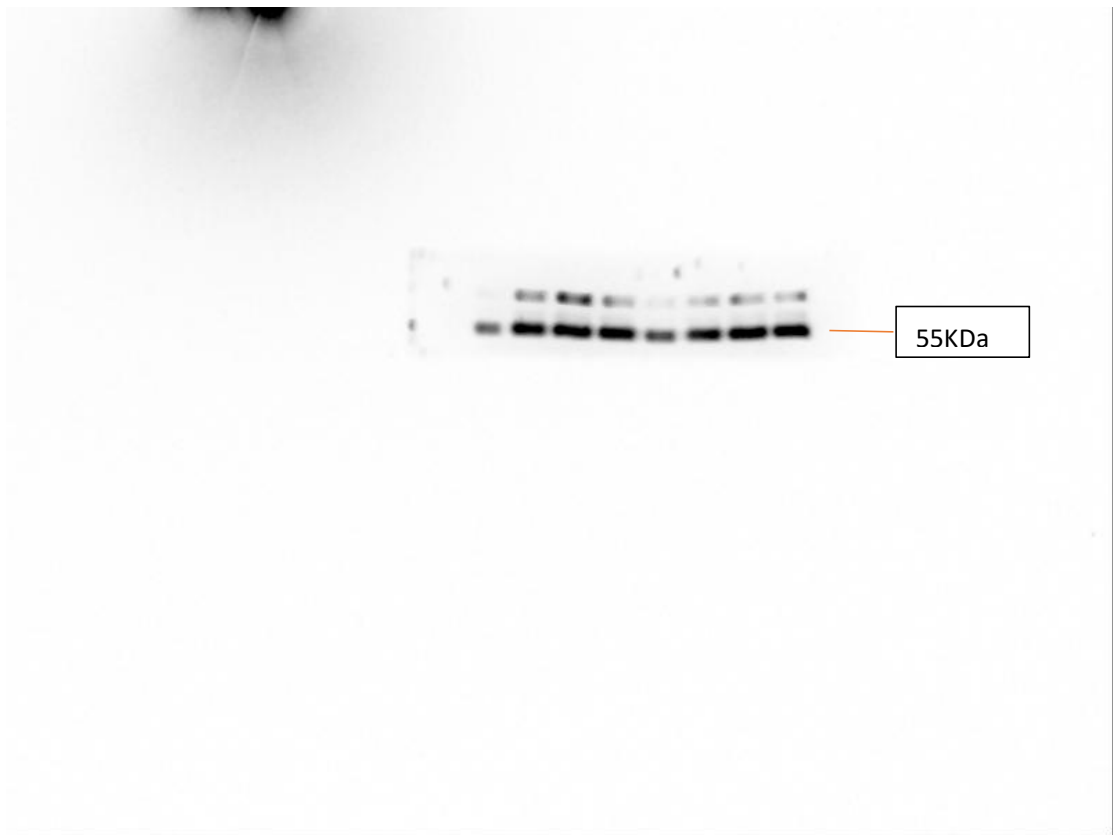

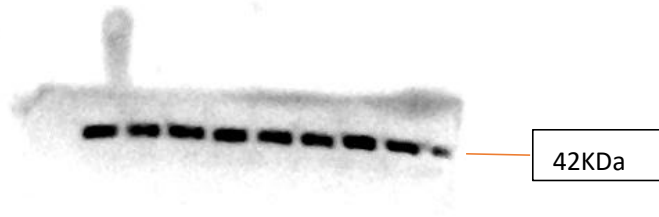

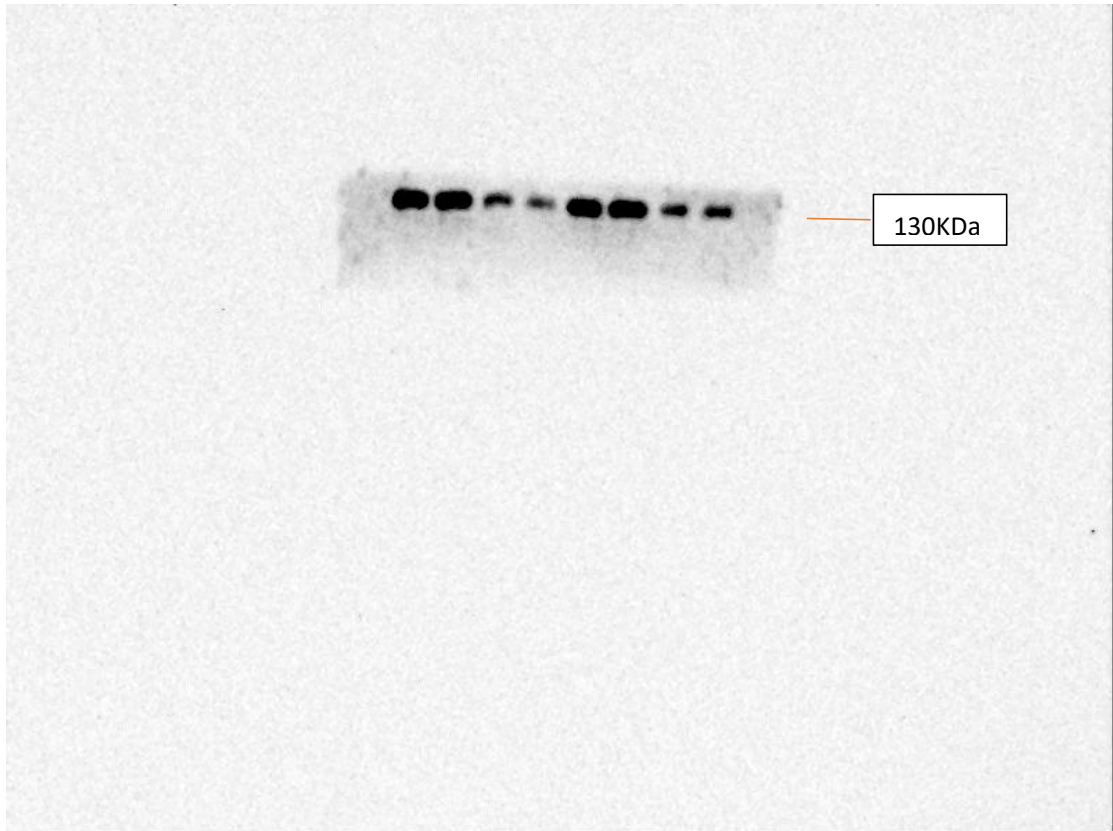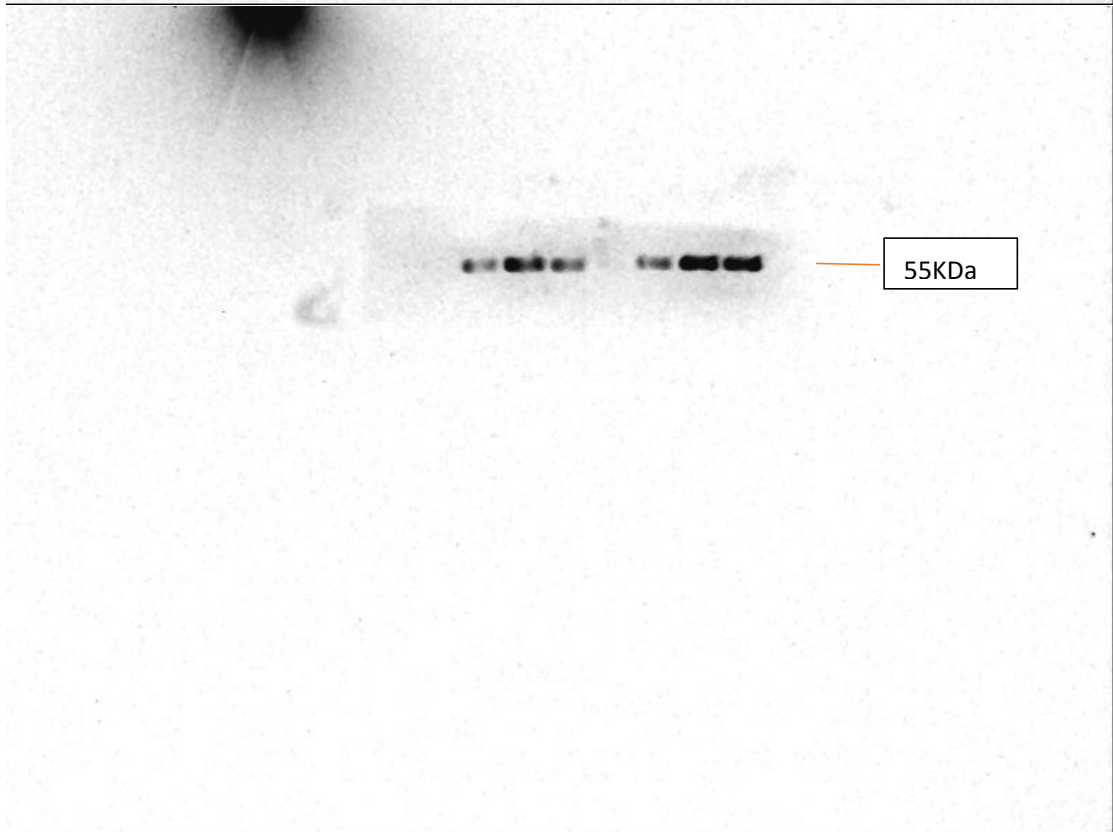

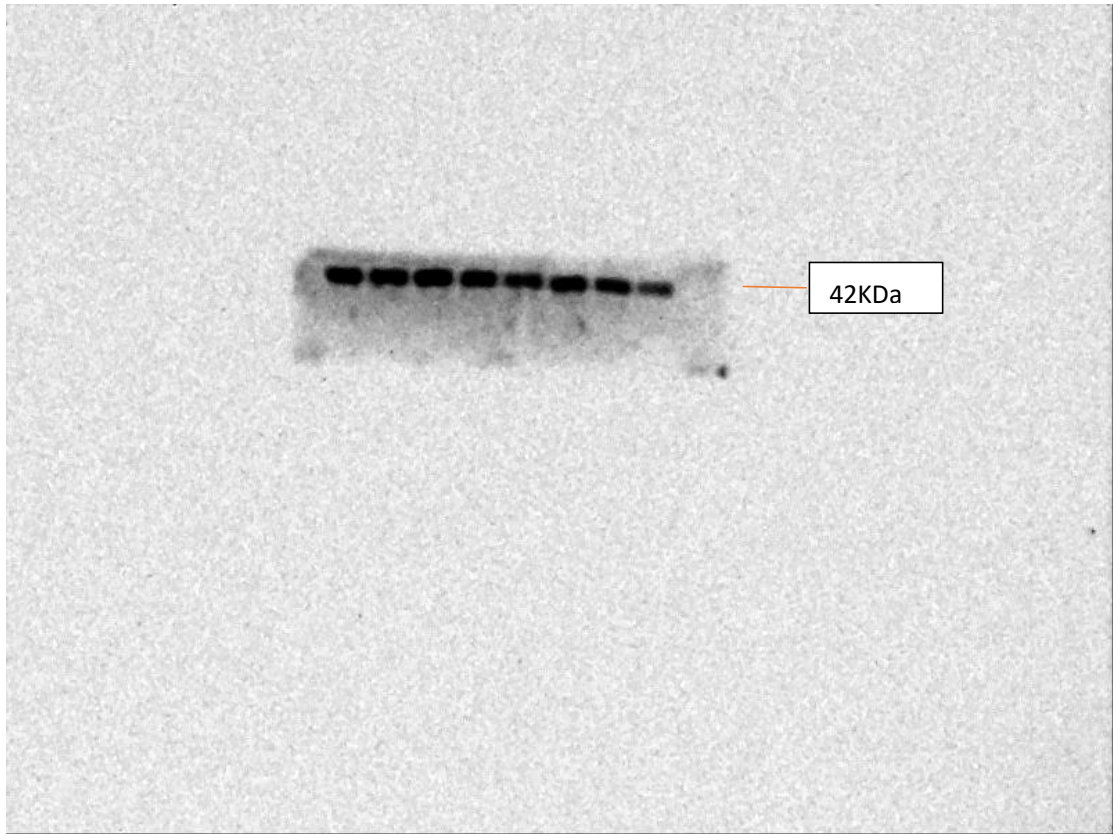

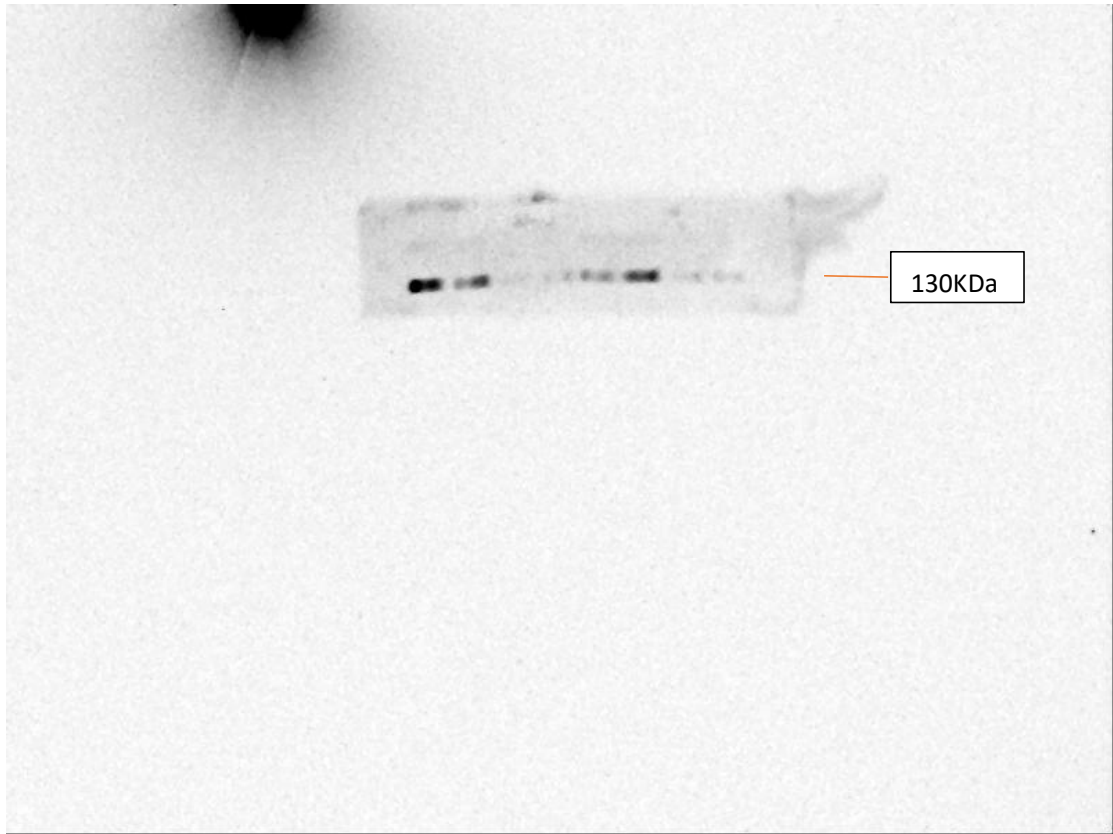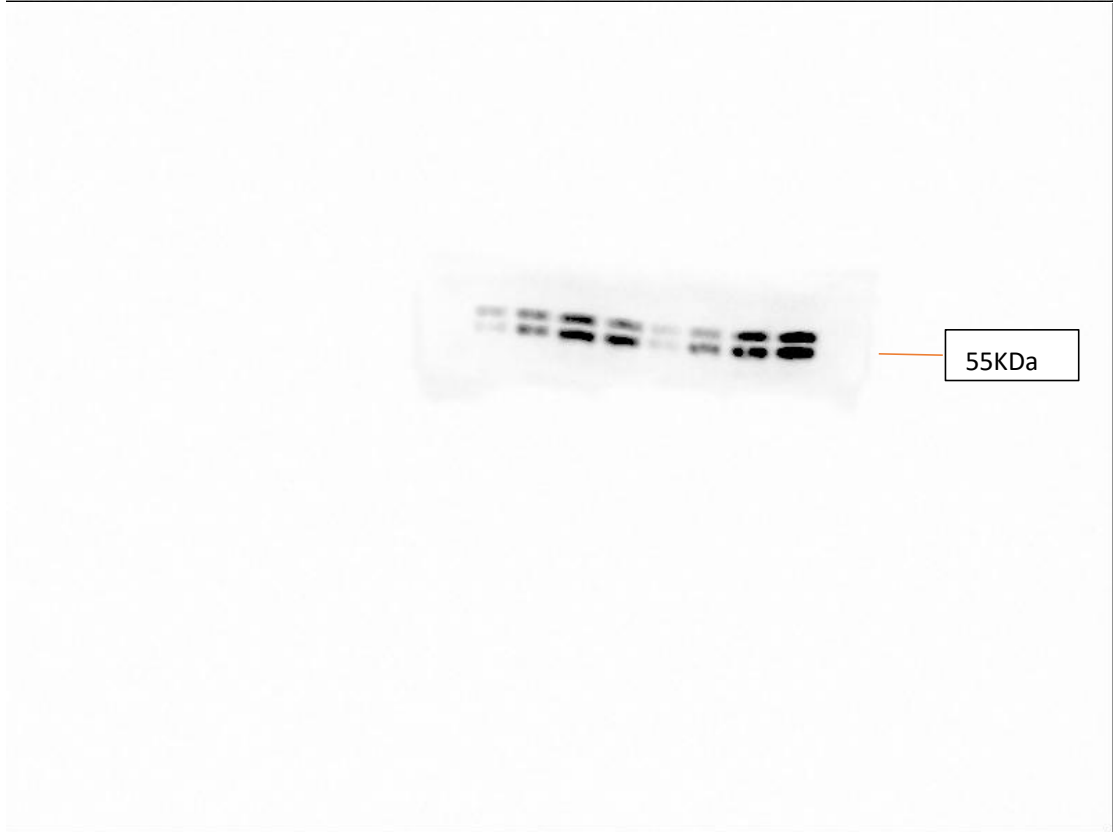

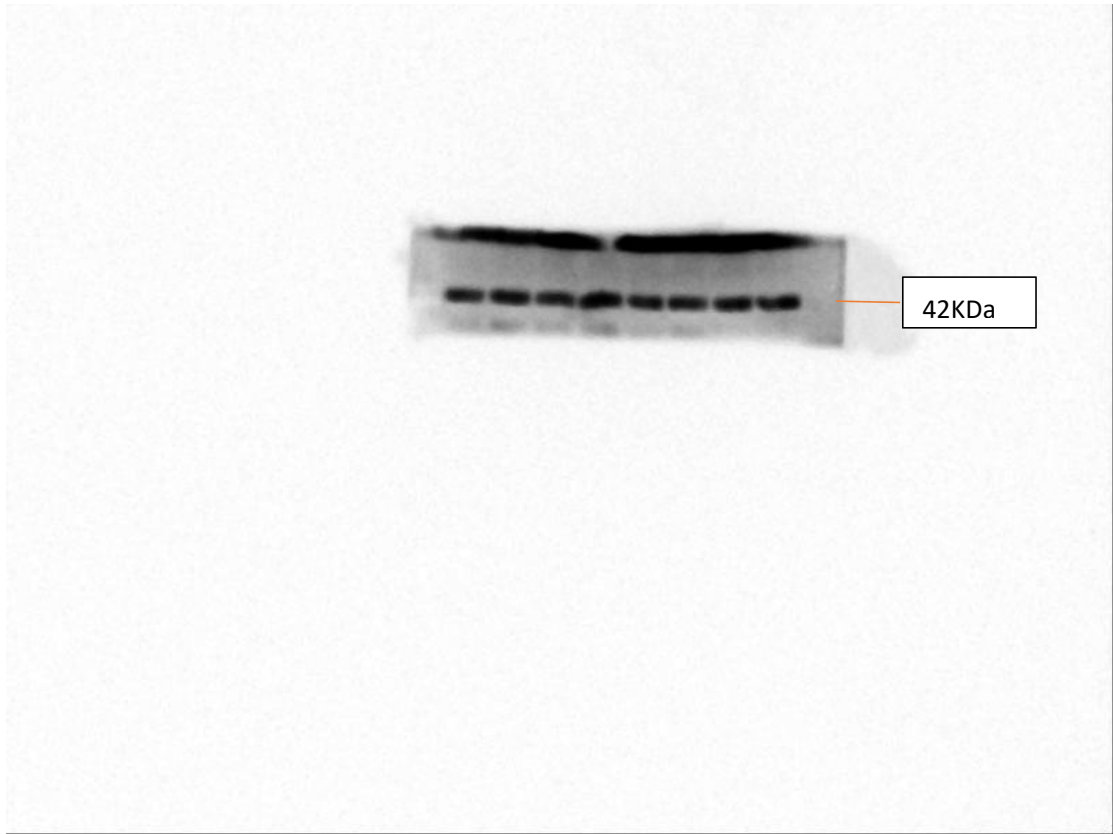

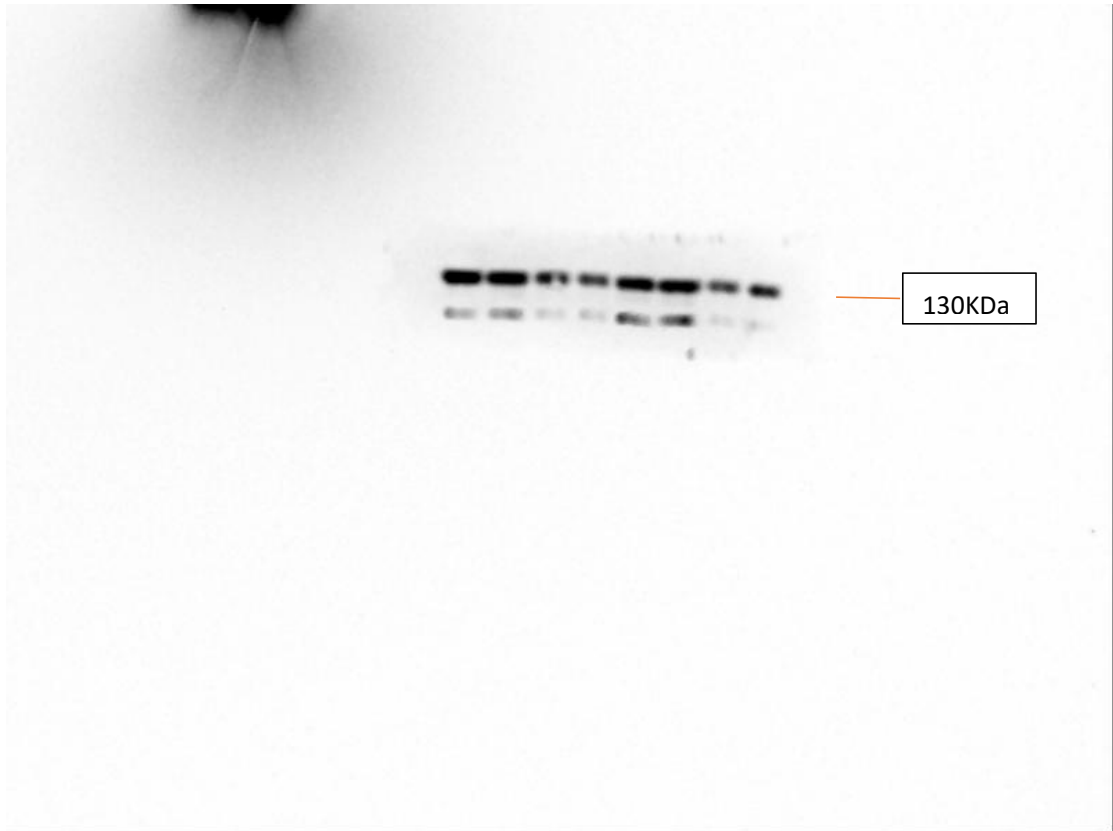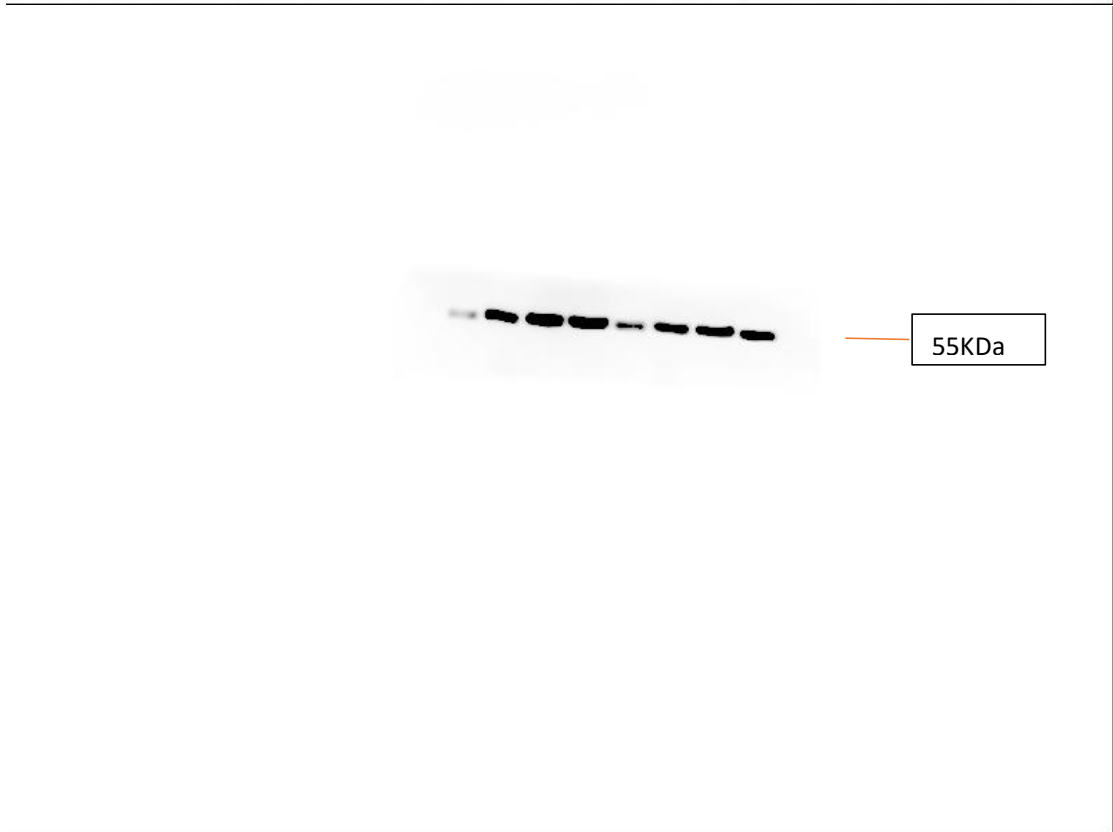

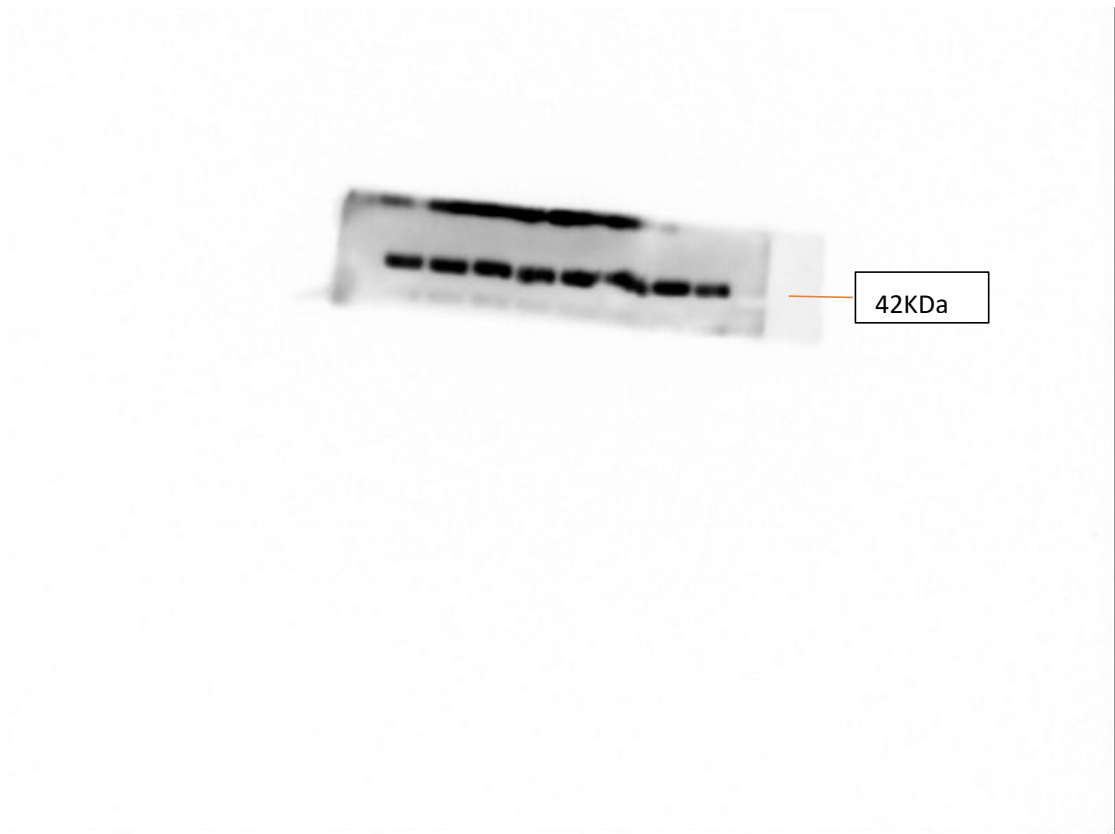

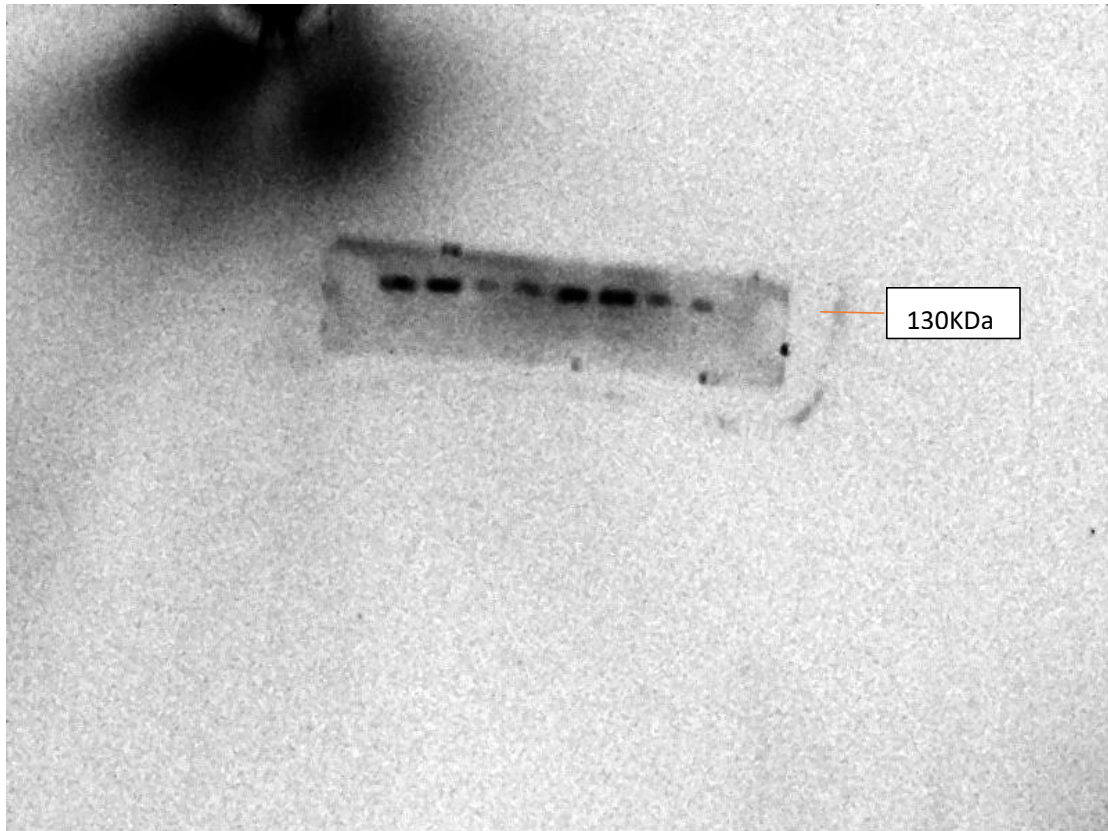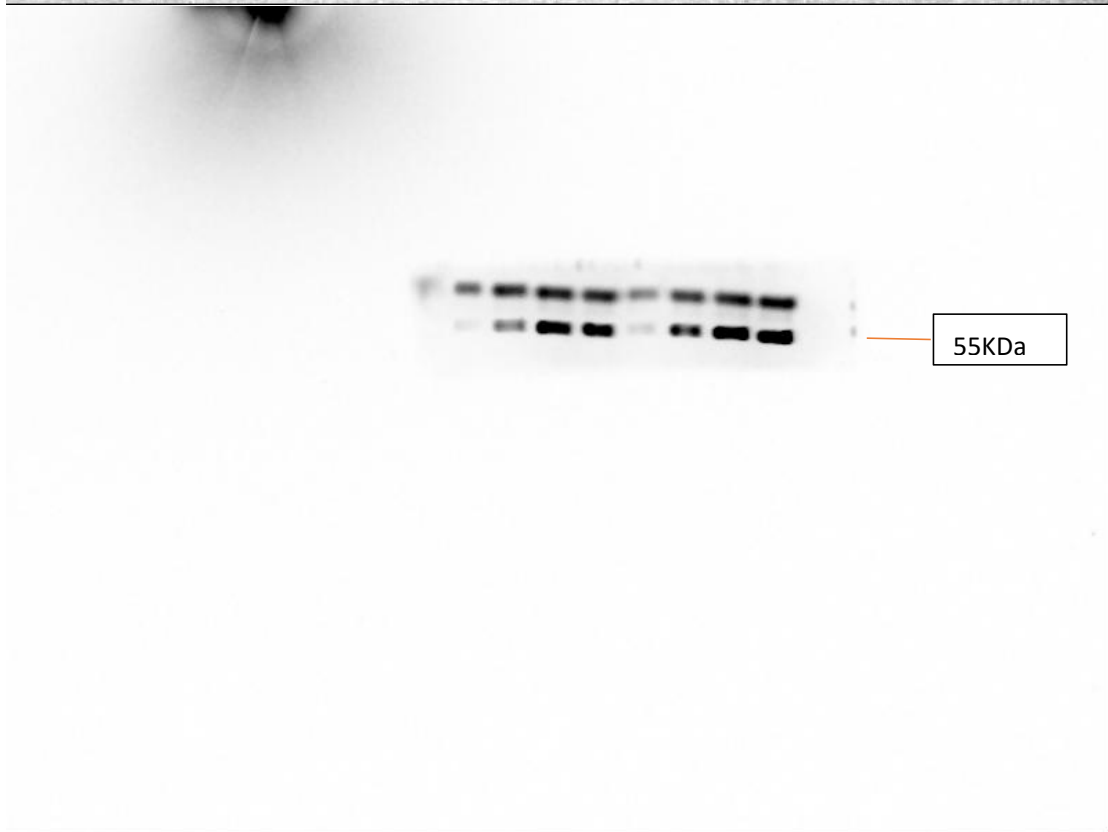

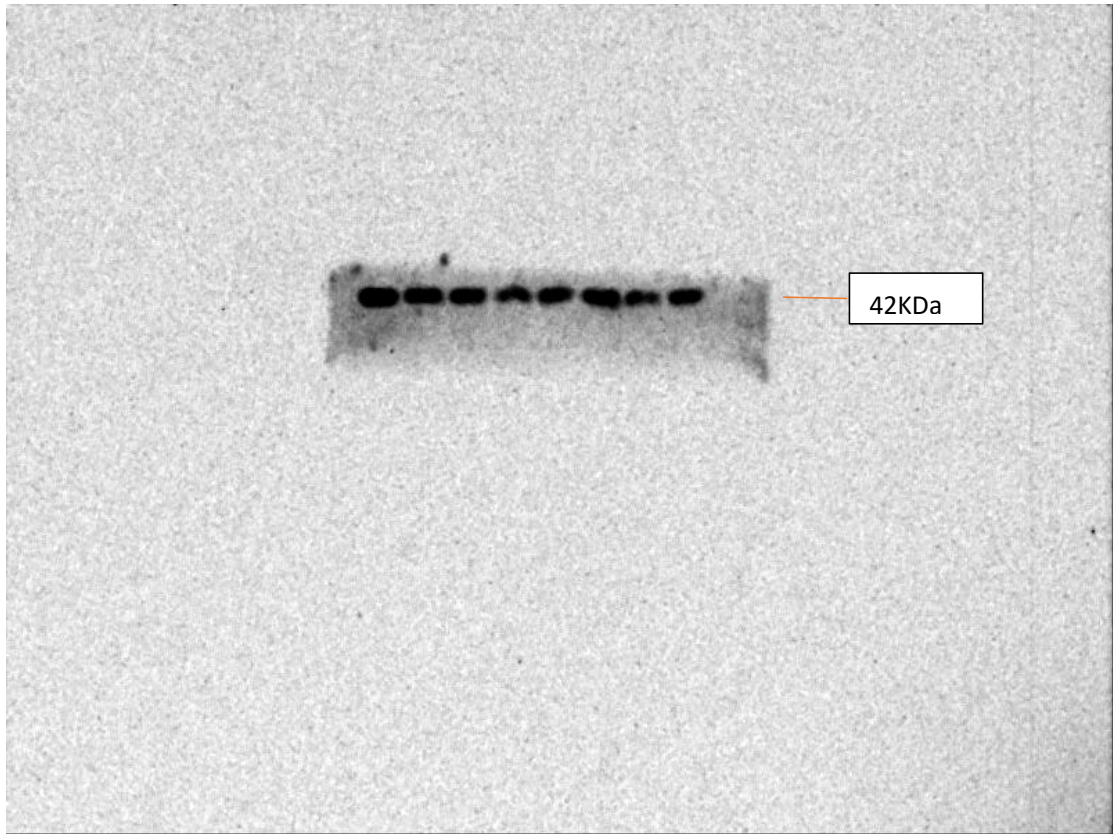

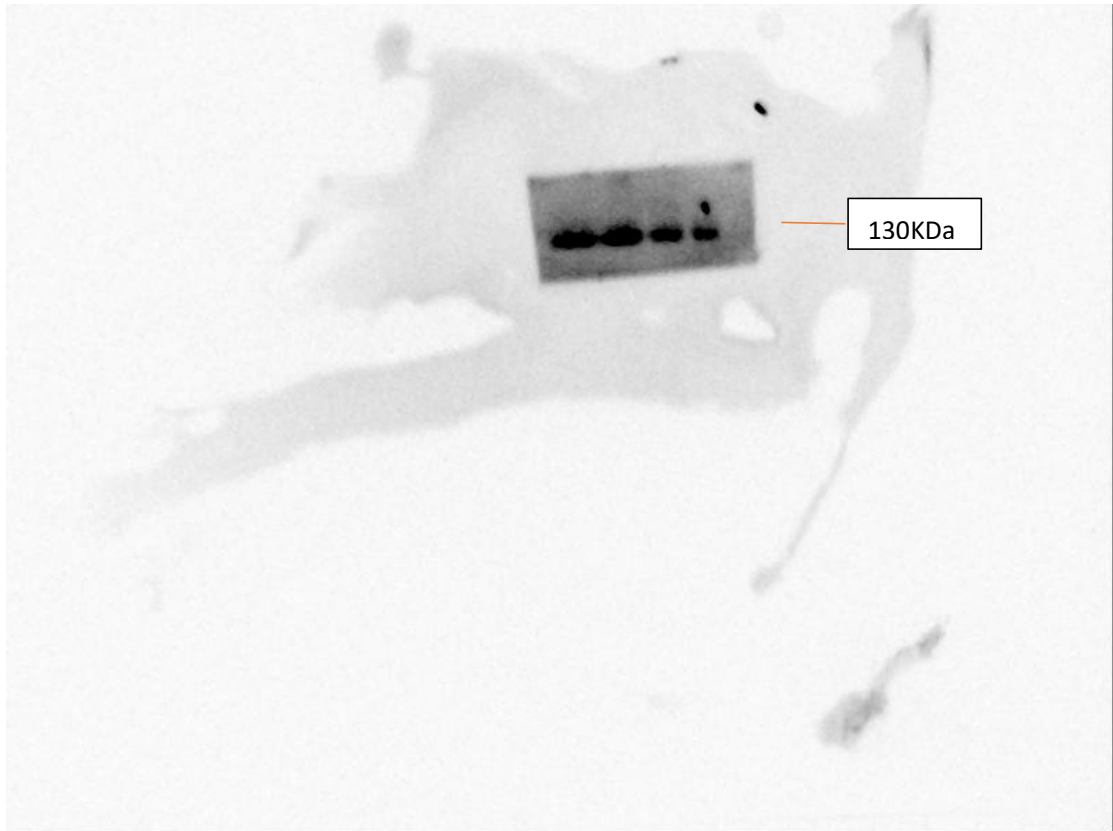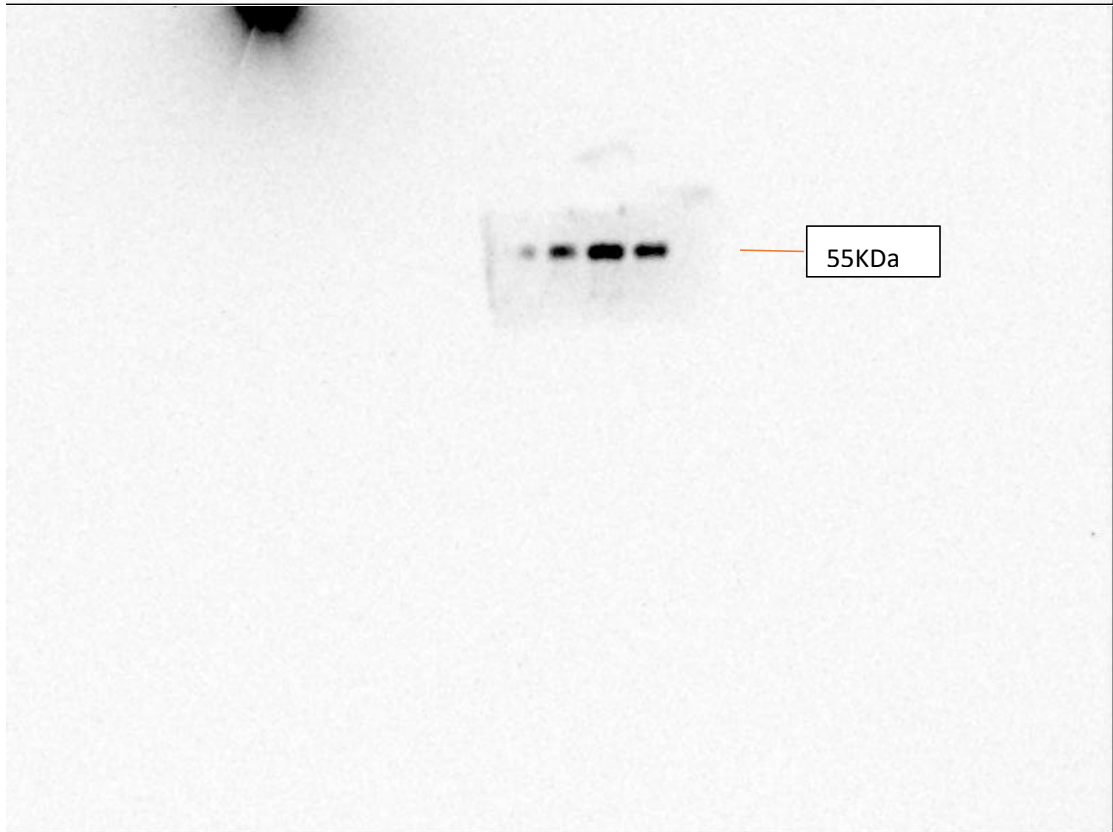

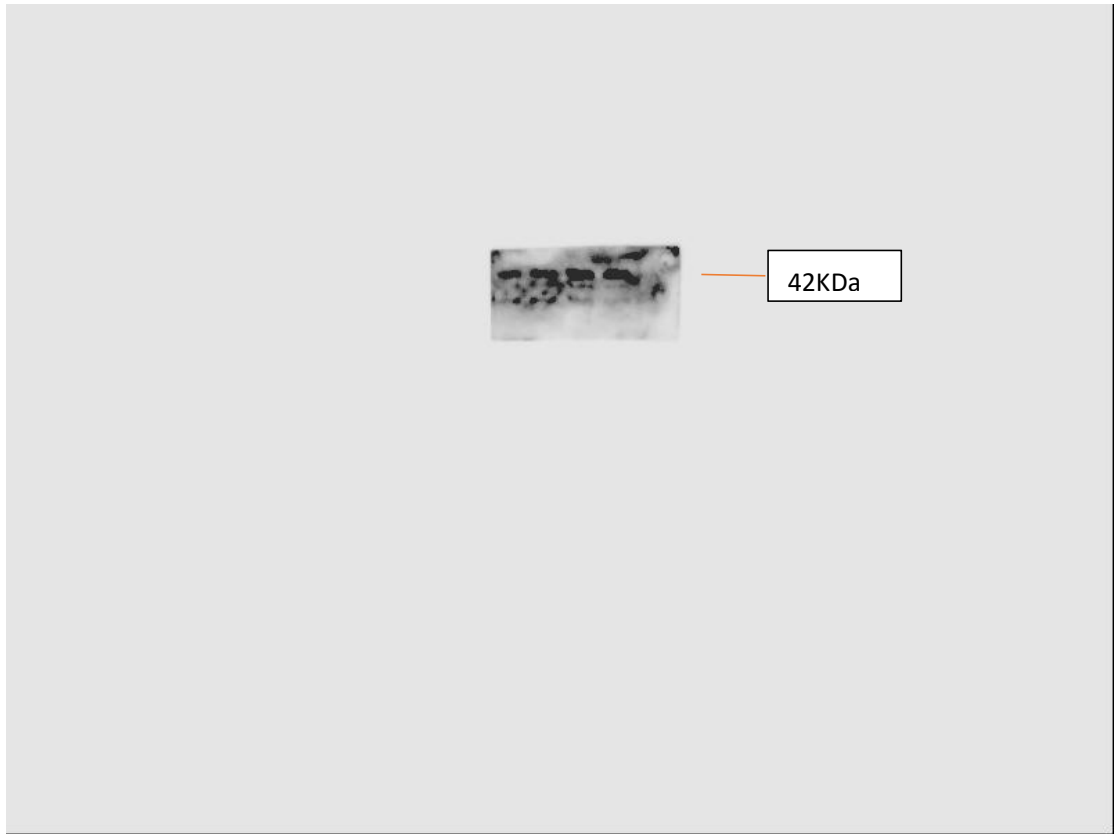

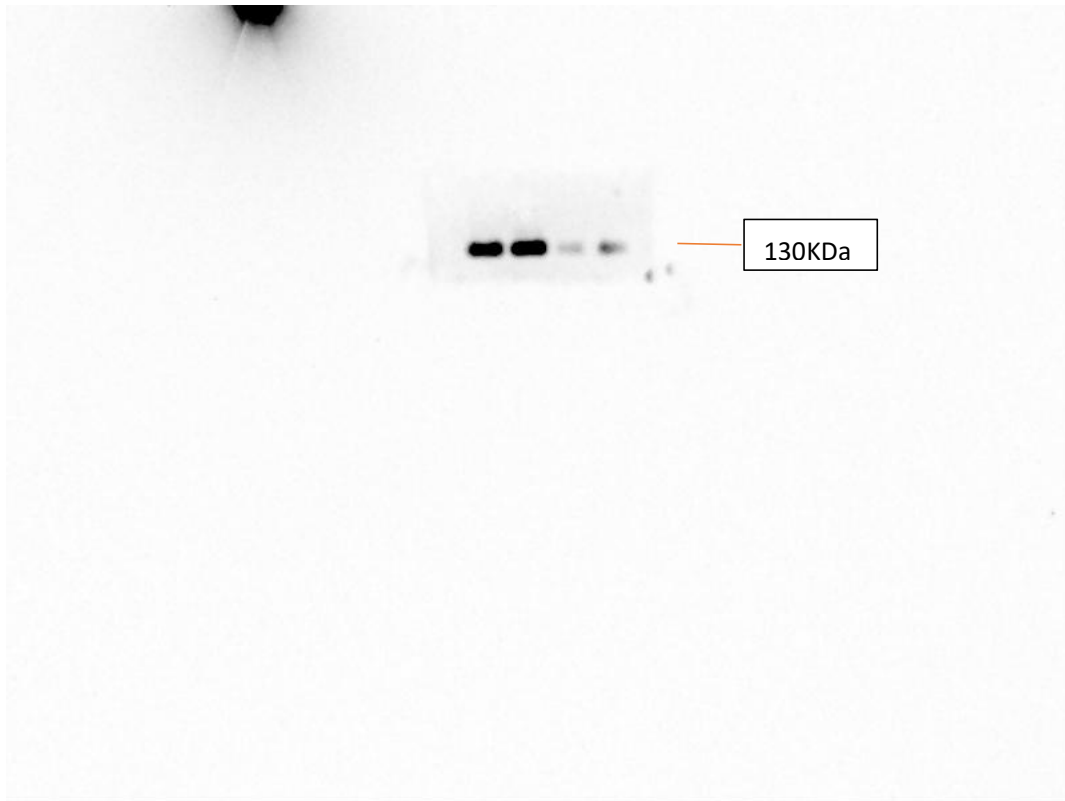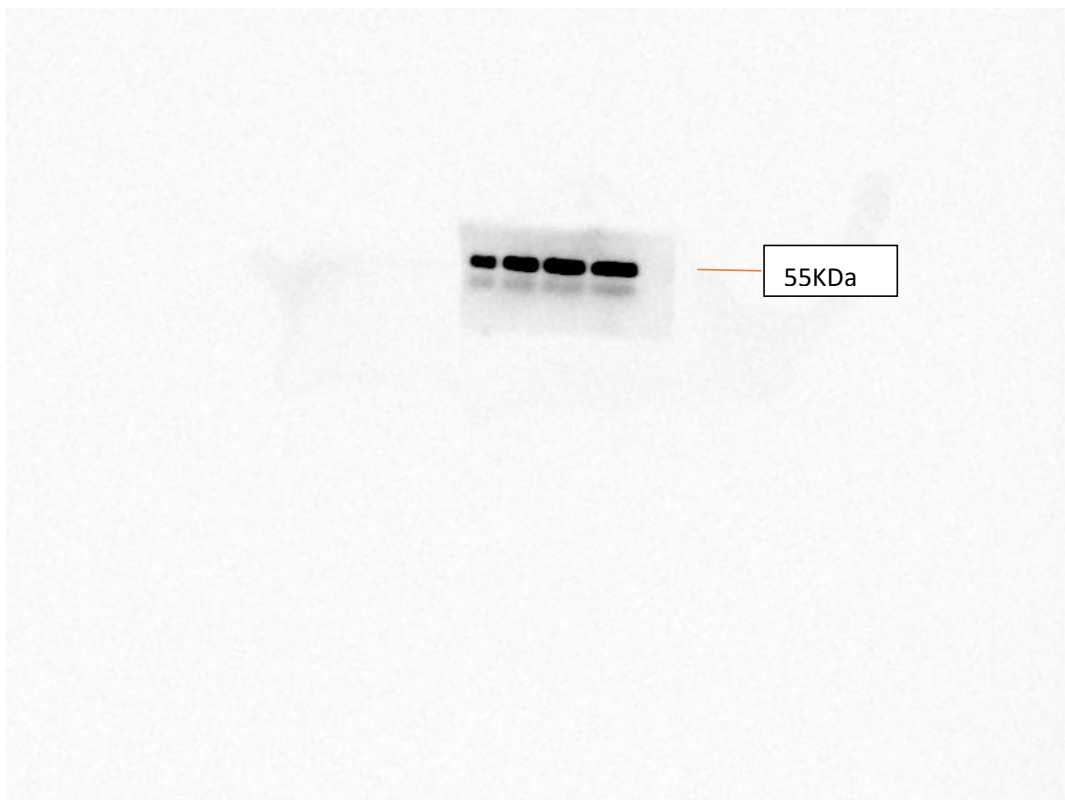

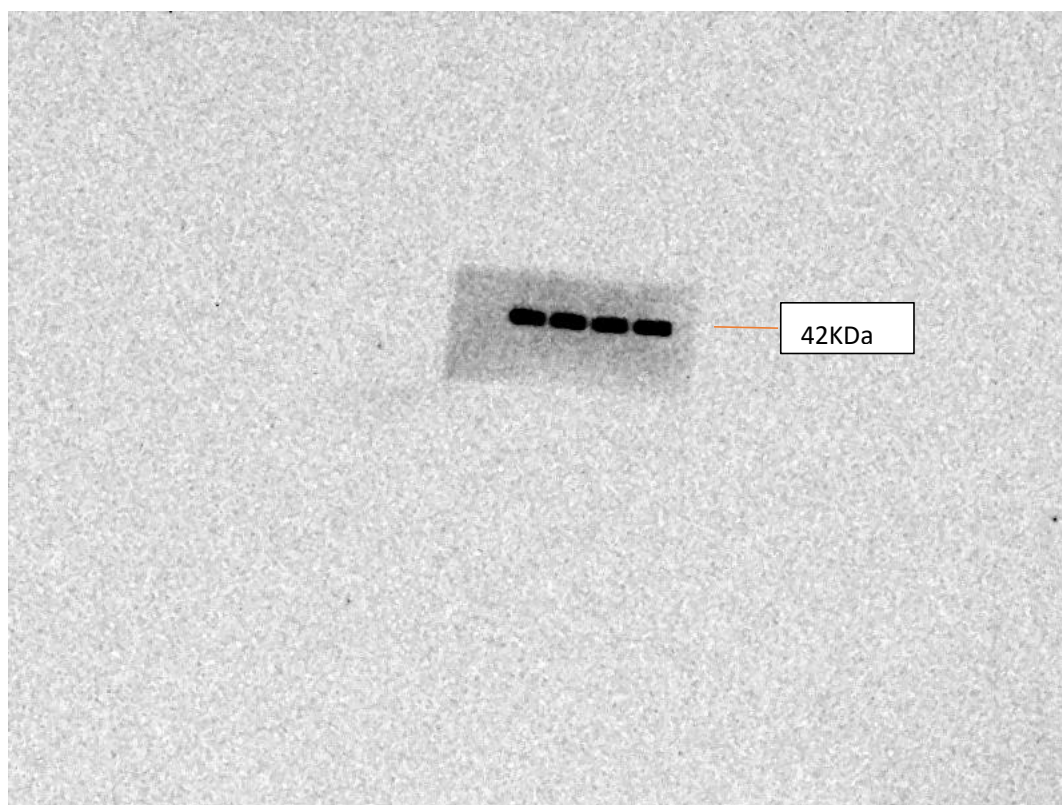

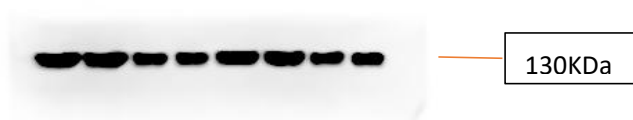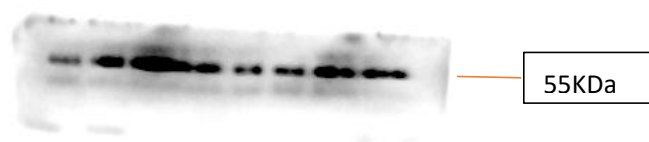

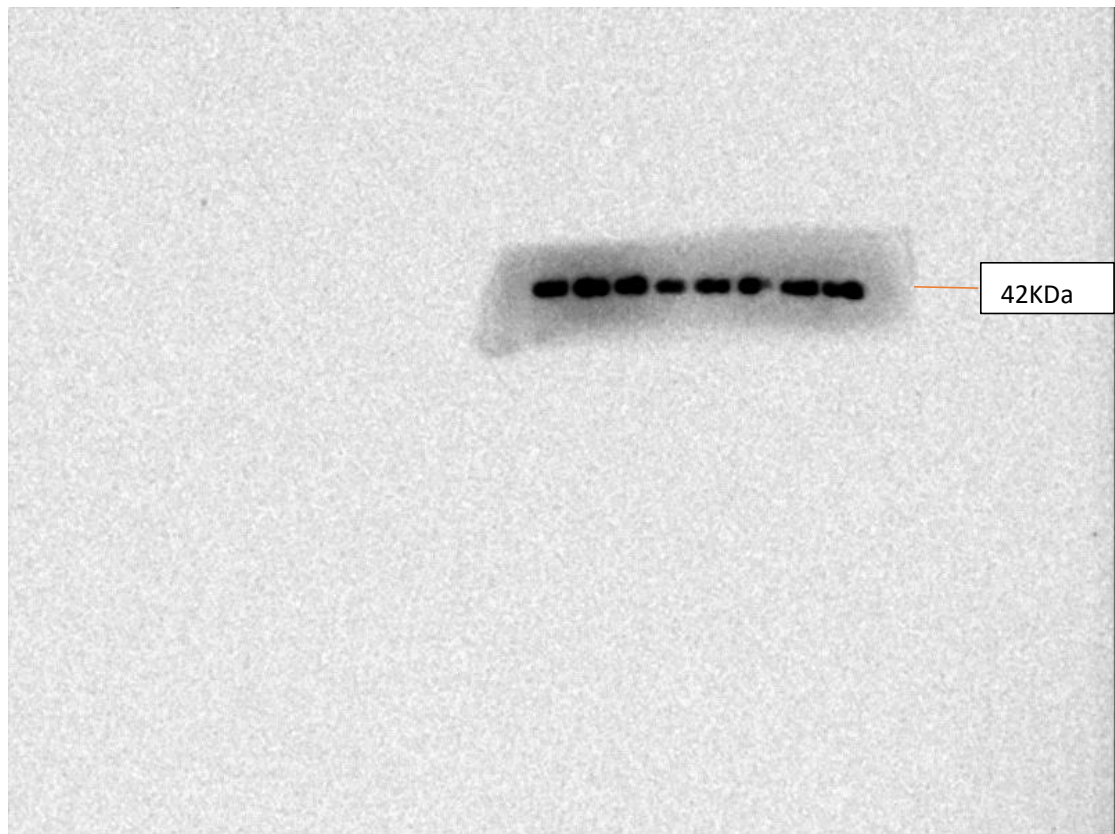

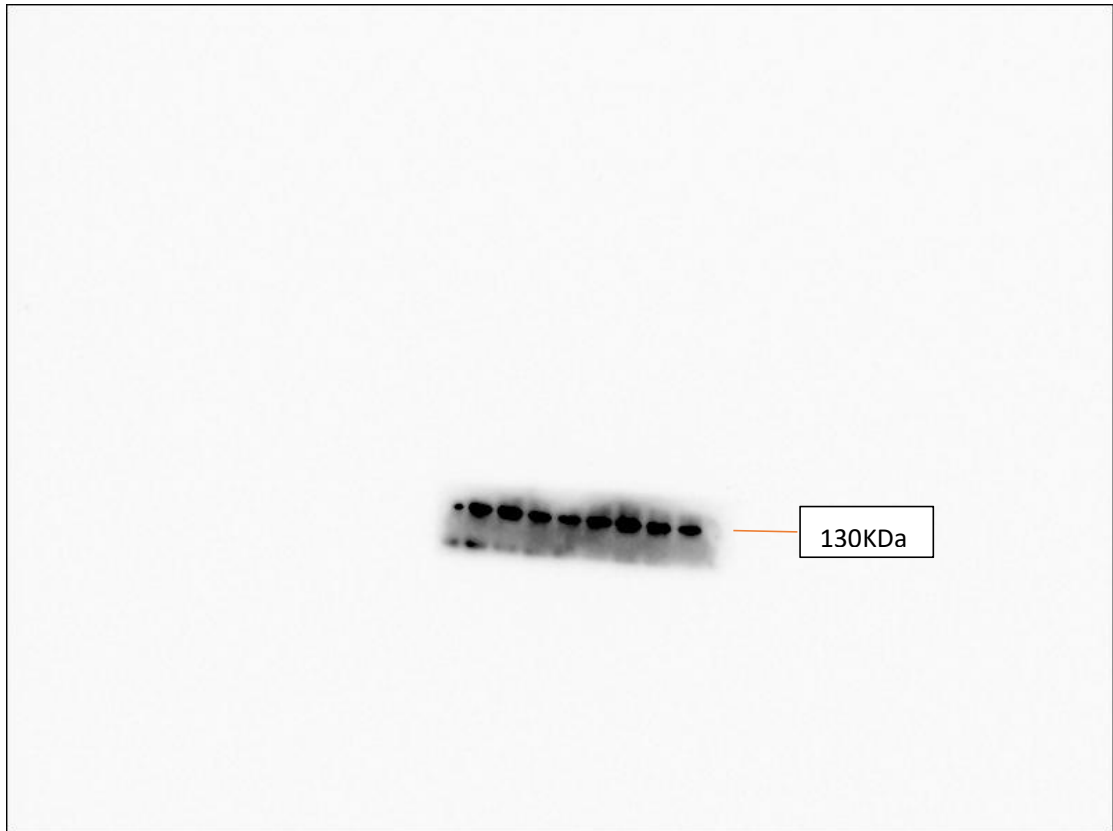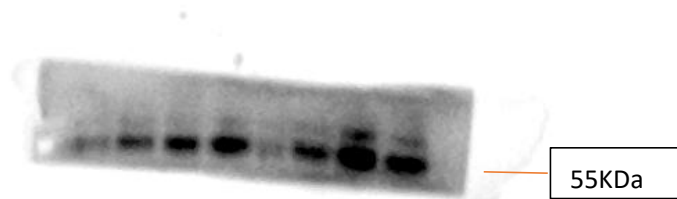

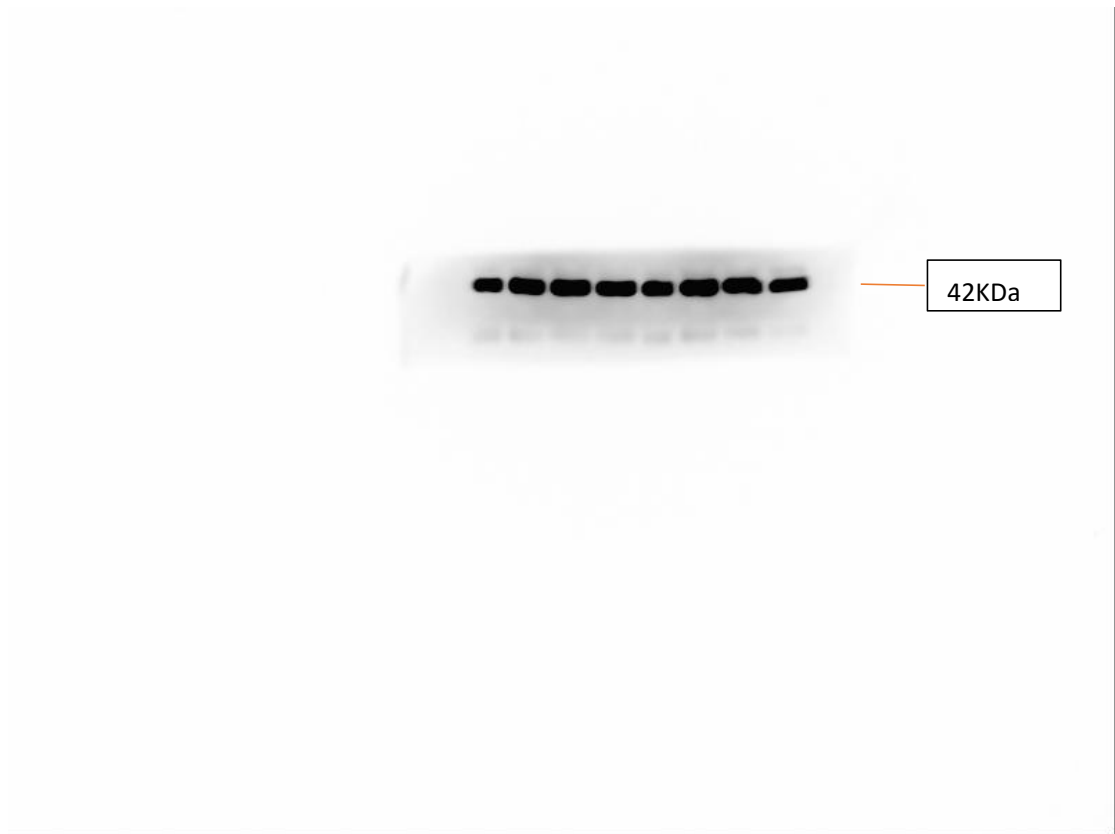

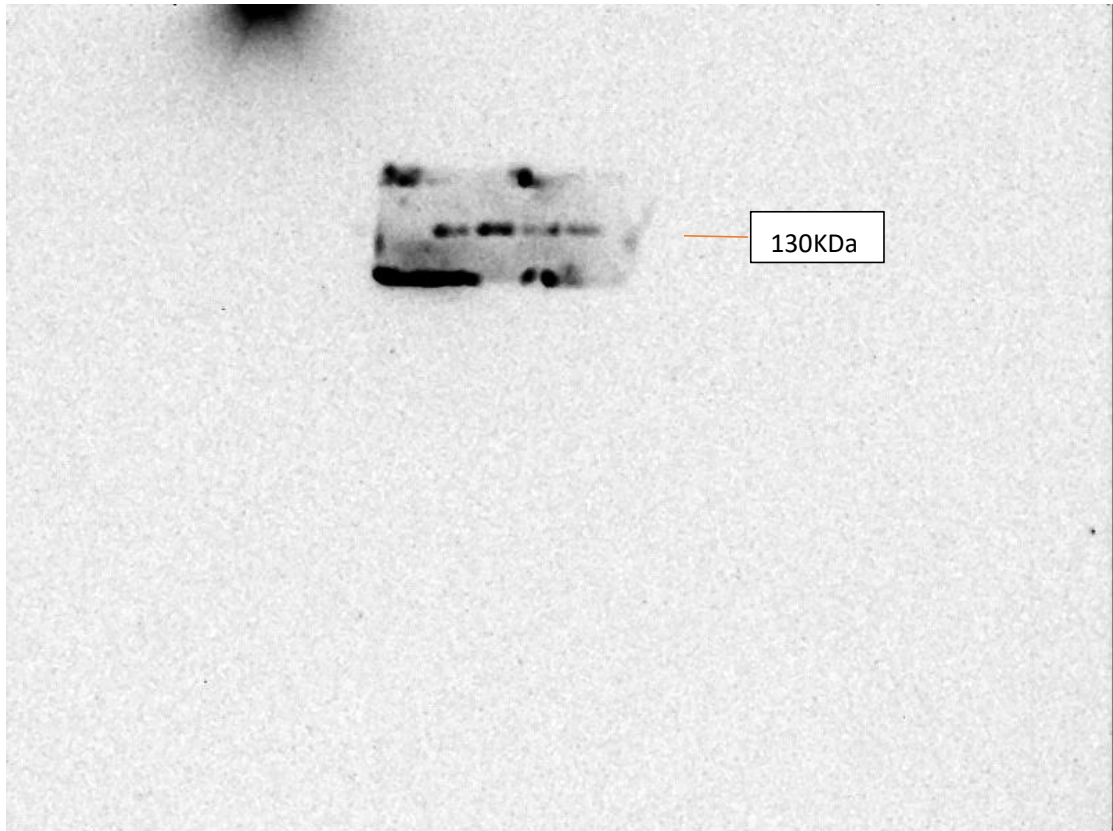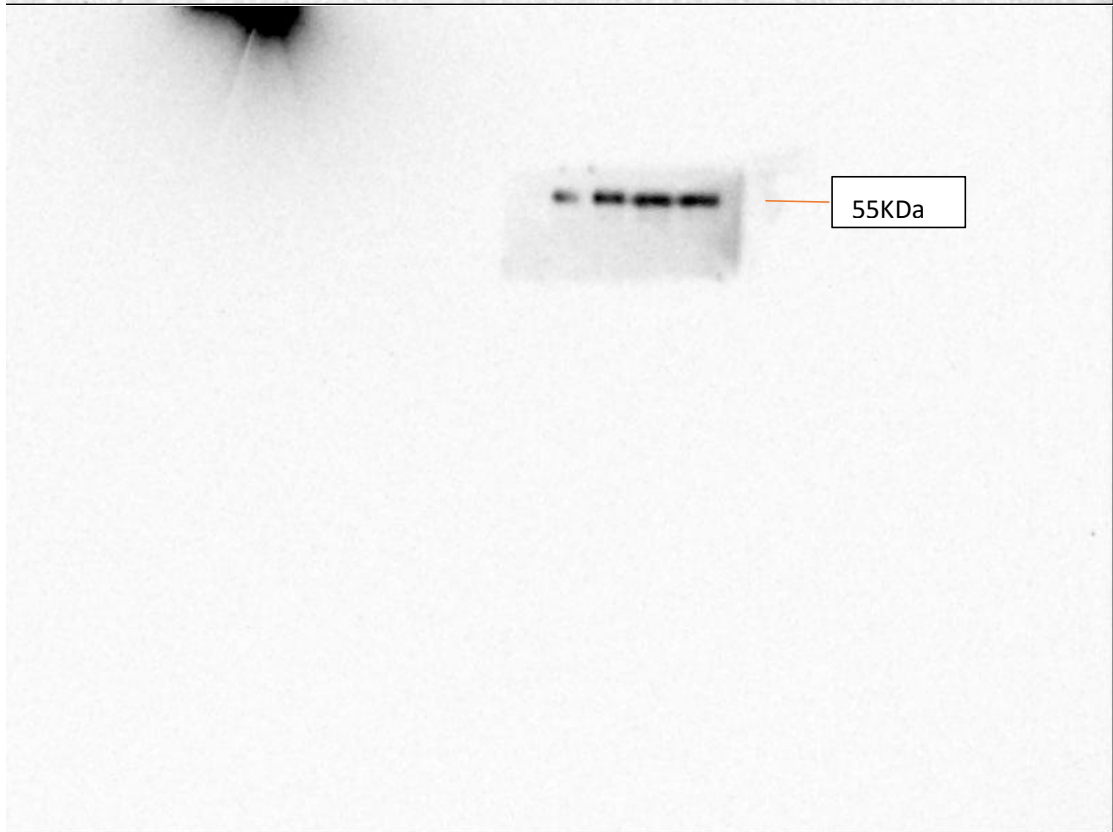

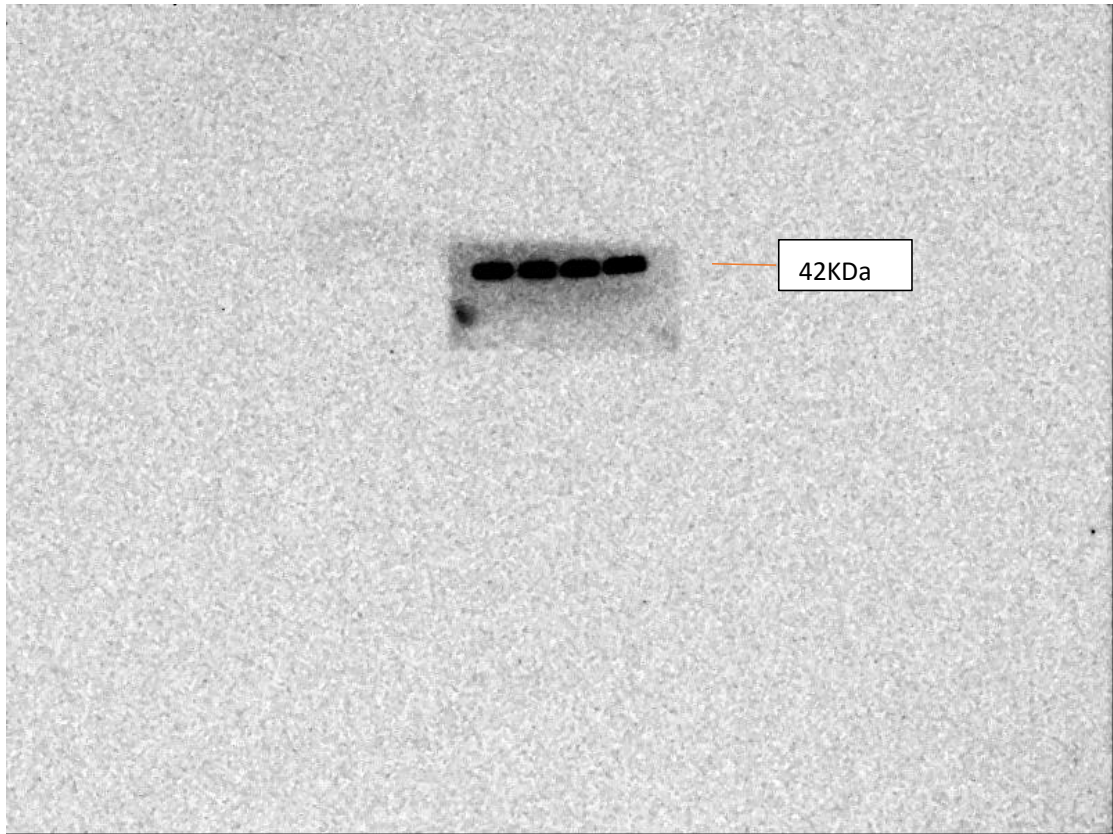

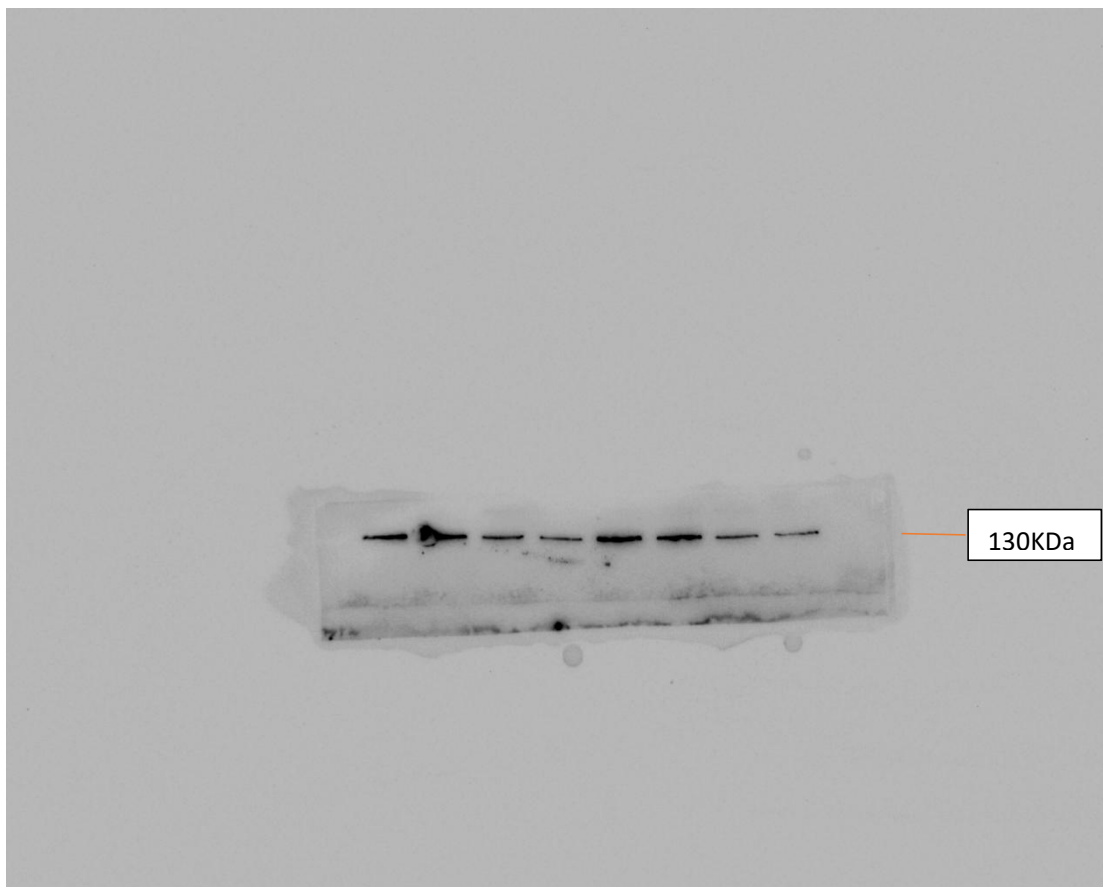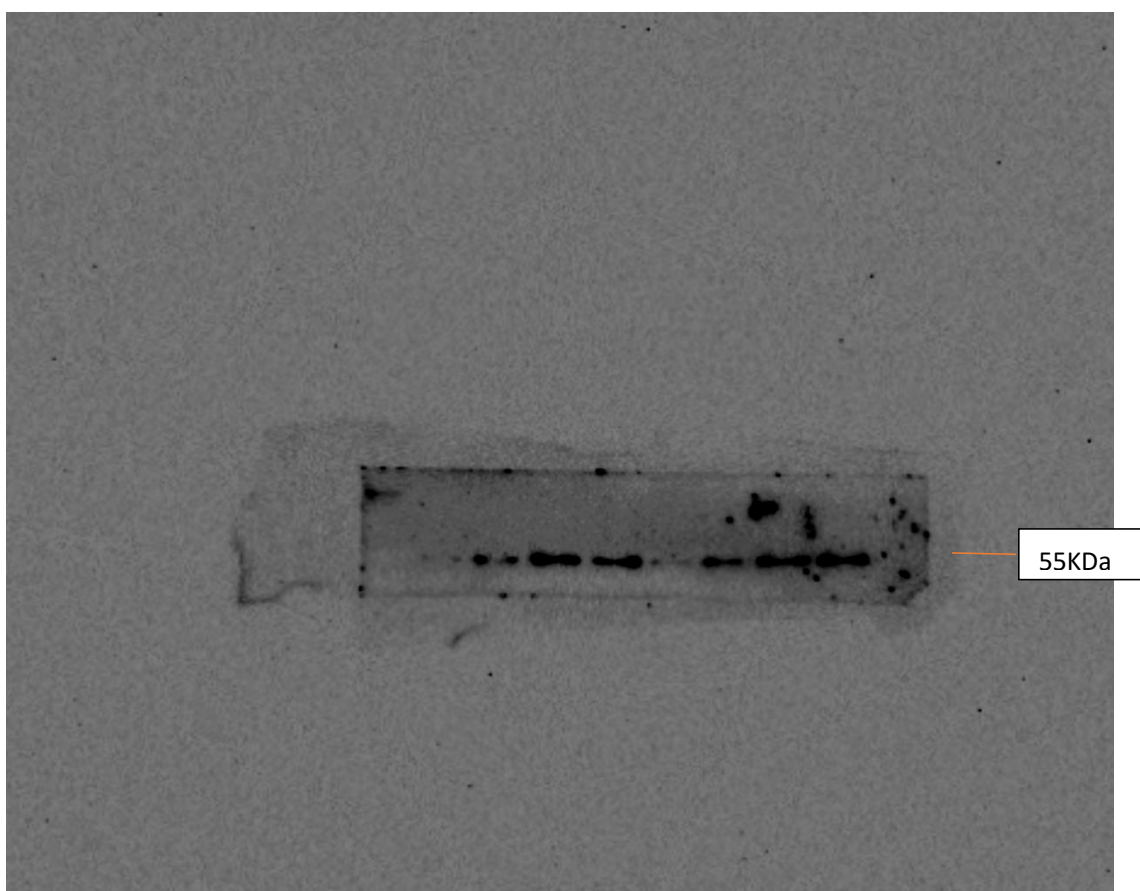

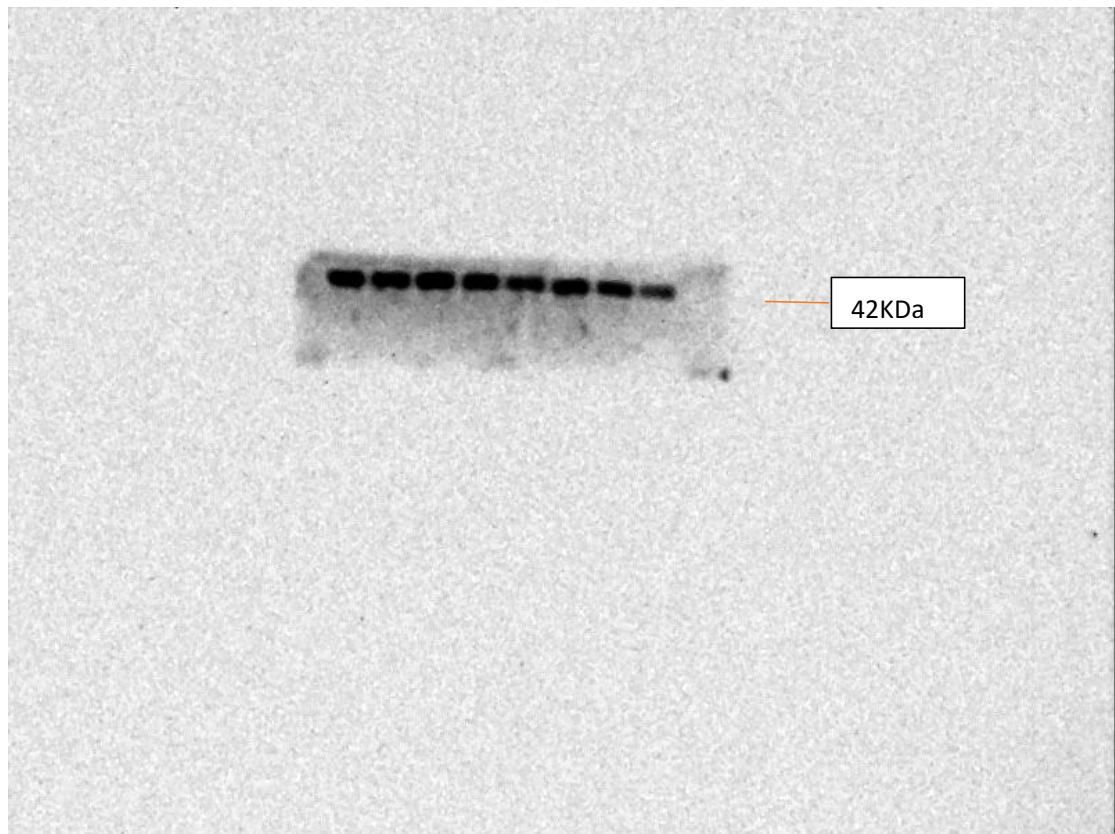

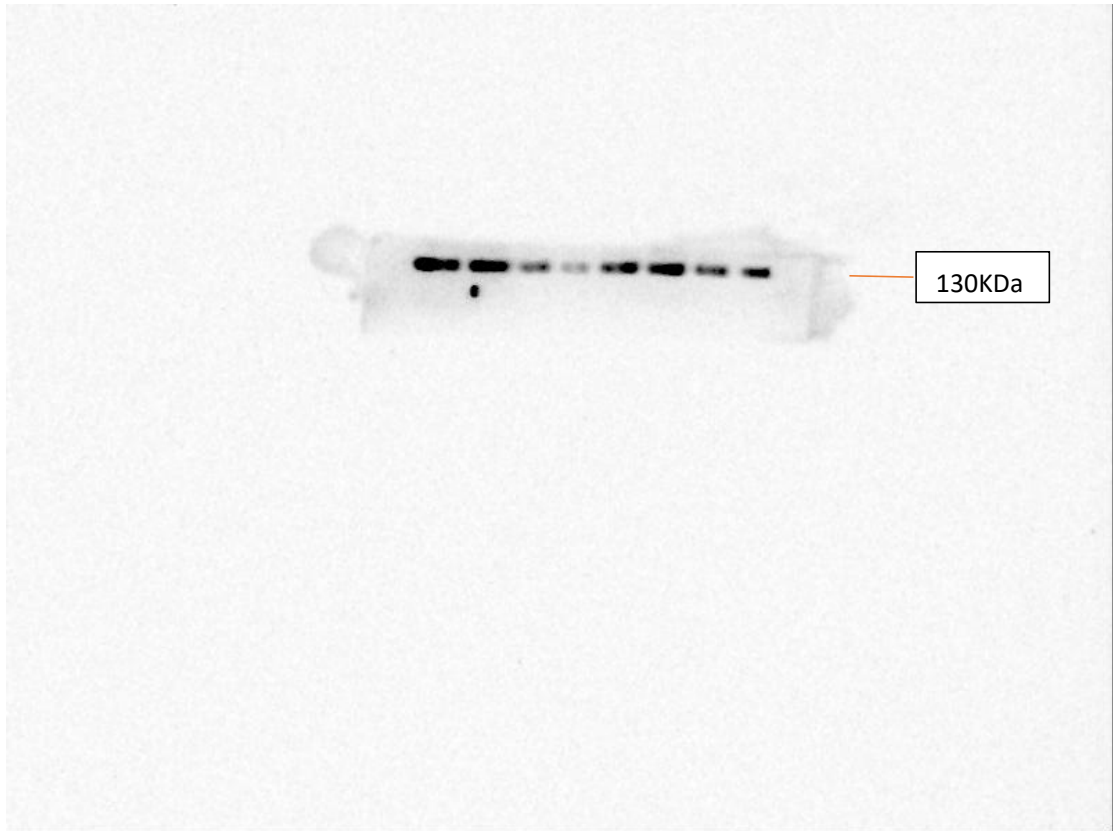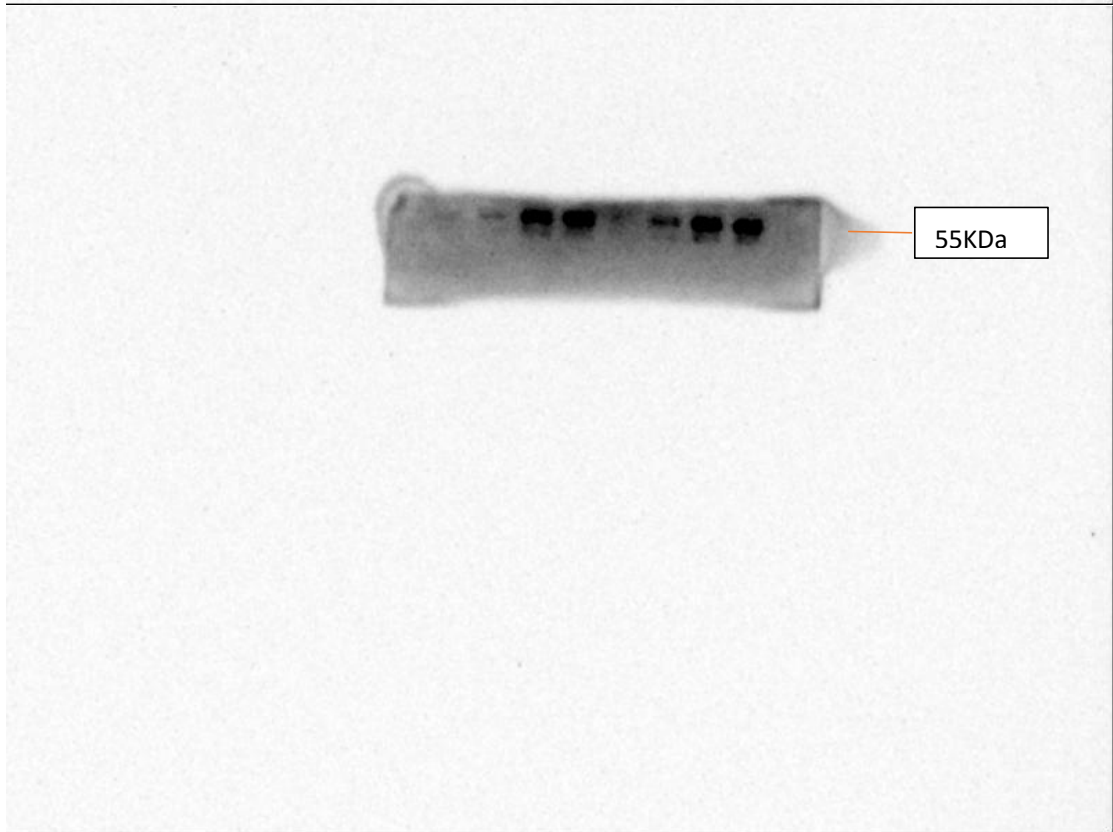

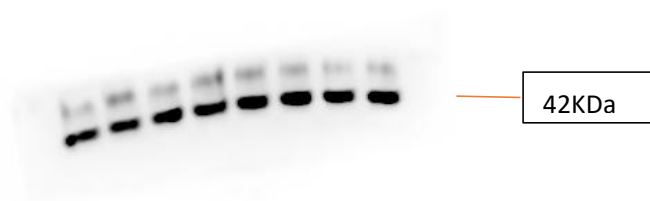

Supplement: Supplementary file 2 — Additional file 2. [file 12891_2022_5427_MOESM2_ESM.pdf]
